# Depletion of CARM1 slows *in vivo* tumorigenicity and rewires developmental programming in glioma stem-like cells

**DOI:** 10.1016/j.celrep.2026.117785

**Published:** 2026-08-07

**Authors:** Dejauwne L. Young, Stephanie Stransky, Maria G. Molero, Erika Yamashita, Shivangi Pande, Vivek Kumar, Saurabh Singh, Jennifer Aguilan, Ronald Cutler, Wade Koba, Joseph D. DeAngelo, Jacob S. Roth, Jinghang Zhang, Tianfang He, Beata Malachowska, Justin Vercellino, Brett I. Bell, David Shechter, Philip J. Tofilon, Richard E. Phillips, Jinan Behnan, Chandan Guha, Simone Sidoli

**Affiliations:** 1 Department of Biochemistry, Albert Einstein College of Medicine, Bronx, NY 10461, USA; 2 Department of Radiation Oncology, Albert Einstein College of Medicine, Bronx, NY 10461, USA; 3 The Leo M. Davidoff Department of Neurological Surgery, Albert Einstein College of Medicine, Montefiore Medical Center, Bronx, NY, USA; 4 Marilyn and Stanley M. Katz Institute for Immunotherapy for Cancer, Department of Microbiology & Immunology, Albert Einstein College of Medicine, Bronx, NY, USA; 5 Ruth L. and David S. Gottesman Institute for Stem Cell and Regenerative Medicine Research, Albert Einstein College of Medicine, Bronx, NY, USA; 6 Institute for Onco-Physics, Department of Radiology, Albert Einstein College of Medicine, Bronx, NY, USA; 7 Department of Microbiology & Immunology, Albert Einstein College of Medicine, Bronx, NY, USA; 8 Radiation Oncology Branch, National Cancer Institute, Bethesda, MD 20892, USA; 9 Department of Neurology, The Perelman School of Medicine, University of Pennsylvania, Philadelphia, PA 19104, USA; 10 Lead contact

## Abstract

Glioma stem-like cells (GSCs) exploit developmental signaling programs that contribute to glioblastoma heterogeneity and therapy resistance. Here, we define a role for the arginine methyltransferase coactivator-associated arginine methyltransferase 1 (CARM1) in regulating GSC lineage state and survival signaling. CARM1 depletion slows GSC growth, increases apoptosis, alters histone post-translational modifications, and shifts transcriptomic and proteomic profiles toward a radial glial-like state. Loss of CARM1 increases NGFR/NTRK signaling and sensitizes GSCs to NTRK and AKT inhibition. Mechanistically, NFIA is a CARM1 substrate, and mutation of NFIA arginine 389 increases NGFR expression, supporting a role for CARM1-dependent NFIA methylation in NGFR repression. In orthotopic xenografts, CARM1 depletion reduces tumor burden and prolongs survival. These findings identify CARM1 as a regulator of GSC developmental programs and NGFR/NTRK-dependent survival.

## In brief

Young et al. show that CARM1 regulates glioma stem-like cell lineage programs. CARM1 loss shifts cells toward a radial glial-like state, increases NGFR/NTRK signaling and inhibitor sensitivity, and reduces tumorigenicity in orthotopic xenografts.

## Graphical abstract

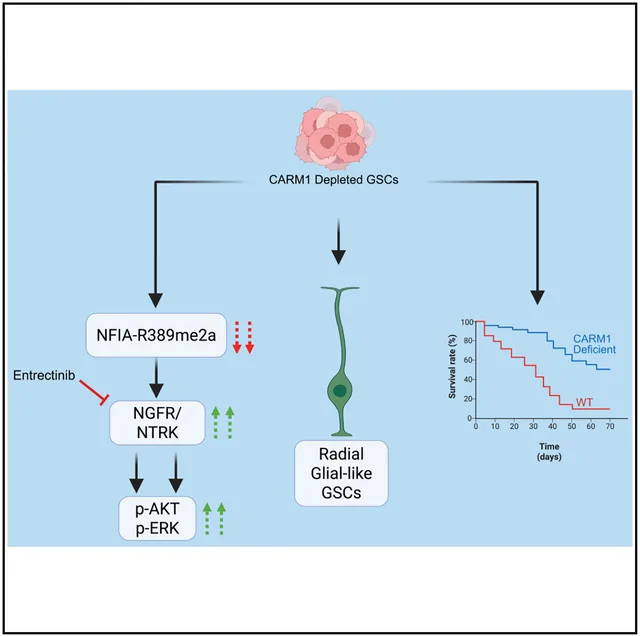

## INTRODUCTION

Glioblastoma (GBM) is the most common malignant primary brain tumor and is universally fatal, with an average survival of 14–16 months from initial diagnosis.^1,2^ GBM aggressiveness is due to a complex interplay of tumor heterogeneity, chemo-radiation resistance, and tumor invasiveness largely driven by glioma stem-like cells (GSCs).^3^ GSCs are characterized by their expression of multiple stem cell markers, capacity to self-renew and differentiate, along with their high tumorigenicity *in vivo*.^4^ Moreover, GSCs rely heavily on epigenetic regulation, defined here as chromatin-associated mechanisms, including histone post-translational modifications (PTMs) and chromatin-modifying enzymes, that influence gene-expression programs without altering DNA sequence, to maintain their stem-like character and evade therapy.^5–7^

One key chromatin-associated regulator of transcription in mammals is protein arginine methylation. Arginine methylation is a vital PTM occurring in mammals and is catalyzed by either type I protein arginine methyltransferases (PRMTs) or type II PRMTs.^8^ Type I PRMTs include PRMT1/2/3/4/6/8 which are responsible for catalyzing asymmetric dimethylarginine (ADMA) and type II PRMTs including PRMT5/9 which catalyze symmetric dimethylarginine (SDMA).^9^ Arginine methylation often functions to promote protein-protein or protein-nucleic acid interactions, and when lost can lead to alterations in gene expression or protein/cellular functions.^10^

Arginine methylation specifically plays a critical role in GBM therapy resistance and chromatin regulation. It has been shown that PRMT1 and PRMT5 are upregulated in GBM and can regulate vital processes such as proliferation, splicing, senescence, and stemness.^8,11–13^ A member of the type I PRMT family that remains under-investigated in GBM is coactivator-associated arginine methyltransferase 1 (CARM1)/PRMT4. CARM1 specifically deposits ADMA marks on histones and chromatin-modulating proteins to exert regulation on transcription and RNA processing.^14^ During embryogenesis, CARM1 has been shown to be involved in regulating cell fate decisions.^15,16^ However, how CARM1 alters developmental signaling and cell fate in GBM remains unknown. Since CARM1 is overexpressed in GBM^17^ and is implicated in normal tissue development, targeting CARM1 may be a promising strategy to selectively target the GSC population. To date, it remains unknown how CARM1 impacts GSC character and to what degree these changes might be therapeutically exploited.

Using CRISPR-Cas9 and a multi-omics approach, we show that loss of CARM1 alters the growth of GSCs *in vitro* and *in vivo* and results in widespread changes in various histone PTMs often involved in development, beyond H3R17. We also find that CARM1 regulates developmental signaling changes on both a transcriptomic and proteomic level and represses the radial glial cell lineage in GSCs. Moreover, we observe that loss of CARM1 increases NGFR/NTRK signaling in GSCs and survival dependency on this pathway *in vitro*—likely through CARM1-dependent methylation of NFIA on arginine 389. Overall, our work defines a role for CARM1 in regulating GSC developmental state and NGFR/NTRK-associated survival signaling.

## RESULTS

### CARM1 is overexpressed in glioblastoma and regulates GSCs proliferation and apoptosis

We first wanted to understand the clinical impact of CARM1 in GBM, so we initially analyzed publicly available datasets from The Cancer Genome Atlas (TCGA). We identified that CARM1 is significantly overexpressed in high-grade glioma in comparison to low-grade glioma (Figure 1A), in line with previous findings.^17^ Since CARM1 reduction has been previously reported to slow cell growth in LN229 cells,^18^ we hypothesized that depletion of CARM1 would also slow proliferation of GSCs. As a model system, we used CRISPR-Cas9 along with a negative selection approach^19^ to ablate the CARM1 gene in patient-derived GSCs that were previously characterized^20^ (Figure 1B). We validated CARM1 depletion in two distinct GSCs using western blot (Figures 1C and 1D). We tested GSC cell growth across multiple time points and found that loss of CARM1 slowed GSC cell growth in both cell lines (Figures 1E and 1F). Further, CARM1 expression was modestly elevated in GSCs cultured under stem cell conditions compared to GSCs differentiated for 7 days in serum-containing media (Figure S1). Next, we assessed differences in cell cycle using flow cytometry and found CARM1-depleted cells had a slight reduction in the percentage of cells in G2/M phase, without significant differences in G0/G1 or S-phase (Figure S2). Flow cytometry analysis for apoptosis with Annexin V and propidium iodide (PI) demonstrated that CARM1-depleted GSCs gain a modest increase in apoptosis compared to control cells (Figures 1G and 1H).

### CARM1 regulates widescale transcriptome and histone changes involved in development

CARM1 has been reported to be involved in cell development and differentiation in various organ systems, including the nervous system,^15,21,22^ but its role in developmental signaling in glioma is still unknown. To identify developmental signaling programs influenced by CARM1 in GSCs, we performed RNA-sequencing (RNA-seq) on neural stem cell 11 (NSC11) sgSCR, sgCARM1-1, and sgCARM1-2 cells. Loss of CARM1 causes significant global changes in GSC protein coding genes (adjusted *p* value < .05) with a total of 1,452 differentially expressed genes (Figure 2A). We find significant dysregulation of key developmental genes such as NGFR, POU3F1 (OCT6), GFAP, CHI3L1, and ARX (Figure 2A; Table S1). Moreover, using Gene Ontology (GO) gene set enrichment analysis (GSEA), we find that loss of CARM1 results in downregulation of transcriptional pathways involved in both stem cell maintenance and chromatin organization (Figure 2B; Table S1). In line with our results, CARM1 has been shown to be involved in histone arginine methylation, which can impact chromatin organization.^23,24^ Interestingly, loss of CARM1 results in reduced gene expression of multiple key enzymes involved in catalyzing or removing histone PTMs besides those involved in histone arginine methylation (Figure 2C). Specifically, we find that loss of CARM1 results in a reduction of key enzymes such as KDM6B, KAT2A, PHF8, and KDM2B (Figure 2C), which have been shown to regulate histone H3K27me3, H3K18ac, H4K20me3, and H3K36me2, respectively. KDM6B, PHF8, and KDM2B are known histone H3/H4 lysine demethylase enzymes that either directly or indirectly remove H3K27me3, H4K20me3, and H3K36me1/2 respectively, while KAT2A is the primary catalytic lysine acetyltransferase of H3K18ac.^25–29^ Since these histone PTMs are often reported to be involved in cell development,^30–34^ and we observe strong differential expression of developmental genes upon CARM1 depletion, we hypothesized that H3K27me3, H3K18ac, and H4K20me3 would be dysregulated in sgCARM1 GSCs compared to sgSCR. To test for alterations in histone PTMs, we isolated histones from sgSCR and sgCARM1 cells followed by liquid chromatography-mass spectrometry (LC-MS) as noted previously.^35^ We observe global upregulation of co-occurring H3K27me3K36me2 and H4K20me3, and downregulation of H3K18ac (Figure 2D), which is directionally in line with the differential expression observed in KDM6B, KDM2B, PHF8, and KAT2A. We also observe a profound loss in H3K79me2, a mark shown to be a transcriptional driver of developmental genes in various cell types, including heart and brain.^36,37^ We did not observe differential expression of the H3K79me2 methyltransferase, DOT1L. In summary, these results demonstrate that loss of CARM1 dysregulates the transcription of protein-coding genes involved in development and chromatin organization and creates global changes in the histone PTM architecture of GSCs.

### CARM1 represses radial glial cell proteomic signature in glioma stem-like cells

Since gene expression may not always correlate to protein expression, we set out to test if developmental changes were also observed on the protein level as this would give further evidence that CARM1 regulates GSC development processes. To test this, we performed global proteomic analysis via LC-MS of NSC11 sgSCR and sgCARM1 GSCs. In support of our transcriptomic findings, we identified that loss of CARM1 drives differential expression of key developmental proteins such as NGFR, GFAP, PDGFRA, NESTIN, NOTCH1, and others (Figure 3A; Table S2). To test for more in-depth functional protein changes, we performed GSEA using C2 Molecular Signatures Database (MSigDB). We found that loss of CARM1 drives increased expression of proteins typically enriched in human radial glial cells compared to neurons^38^ (Figures 3B and 3C; Table S2), which we also observe in our transcriptomics data (Figure S3). Radial glial cells are an early type of neural progenitor cells that arise during embryonic development and possess extensive migratory capabilities and astrocytic characteristics.^39^ Adult human glioma indeed maintains a population of radial glial-like cells which can promote tumor heterogeneity.^40,41^ In addition, GBM cells that adapt new cell lineages often rely on different molecular processes for growth and survival.^42,43^ To understand which molecular alterations might be the most critical to growth and survival of GSCs post-CARM1 depletion, we plotted the RNA-seq and proteomic fold changes of differentially expressed genes in both CARM1 sgRNAs. We found that NGFR was one of the most upregulated genes at both the mRNA and protein level (Figure 3D). After averaging and performing rank analysis of the proteomic and RNA log_2_ fold changes, we observed that NGFR was the 3^rd^ most upregulated gene/protein in our data, just above GFAP—one of the known major astrocytic markers (Figure 3E). NGFR has been shown to be critical in promoting migration during neurodevelopment and tumorigenesis.^44,45^ These results indicate that at both the transcriptomic and proteomic levels, CARM1 naturally represses radial glial cell signatures in GSCs and may become more dependent on NGFR signaling upon CARM1 depletion.

### Loss of CARM1 drives NGFR/NTRK signaling in glioma stem-like cells

NGFR is a transmembrane receptor for neurotrophins such as nerve growth factor and stimulates activity of neurotrophin kinases (NTRK1/2/3).^46^ In addition, NTRK signaling has been shown to be critical to the survival of radial glial cells.^47^ To test if loss of CARM1 leads to upregulation of NGFR/NTRK signaling in GSCs, we applied mass spectrometry-based phospho-proteomics. We observed that loss of CARM1 results in widespread changes in phospho-peptides (Figure 4A). Using Qiagen Ingenuity Pathway Analysis (IPA), we analyzed the most enriched pathways and the predicted upstream regulators and found that NGF/NTRK signaling are both in the top enriched canonical pathways, with NGF ranking as the 2^nd^ highest predicted upstream regulator of the phospho-proteome in CARM1-depleted cells (Figures 4B and 4C; Table S3). NGFR/NTRK signaling leads directly to increased phosphorylated AKT and ERK1/2 and serves as the readout for increased NGFR/NTRK signaling.^48^ In our mass spectrometry analysis, we also observed that loss of CARM1 results in significantly increased phosphorylation of proteins that are phosphorylation targets of NGFR/NTRK signaling, including AKT1 and ERK1/2 (Figure 4D; Table S3). We then further validated that phosphorylated AKT and ERK1/2 are upregulated by removing epidermal growth factor (EGF) and bFGF growth factors for 6 h and performing western blotting. CARM1-depleted GSCs demonstrated increased phosphorylation of the downstream NGFR/NTRK signaling targets AKT1 and ERK1/2 following growth factor starvation (Figure 4E).

Since our results show that loss of CARM1 drives NGFR/NTRK signaling in GSCs and NGFR/NTRK is known to promote differentiation and survival,^48^ we hypothesized that GSCs lacking CARM1 would have increased sensitivity to NTRK inhibition. Currently, there are several NTRK inhibitors that exist, but the compound entrectinib is both FDA approved and brain penetrant,^49–51^ so we use entrectinib as our primary treatment modality. To test if CARM1 loss leads to increased short-term sensitivity of GSCs to NTRK inhibition, we treated GSCs with entrectinib for 96 h and measured cell viability. We found that sgCARM1 GSCs have increased sensitivity to entrectinib compared to sgSCR cells (Figure 4F). Since AKT is also activated by NGFR/NTRK signaling, we also treated GSCs with the FDA-approved AKT inhibitor capivasertib and saw that sgCARM1 GSCs had increased sensitivity to AKT inhibition compared to sgSCR (Figure S4). We next checked long-term proliferative capacity by treating GSCs with 3 μM of entrectinib (IC50 of sgCARM1 cells) using the clonogenic assay. We found that sgCARM1 GSCs were over 4-fold more sensitive to entrectinib compared to sgSCR cells (Figures 4G and 4H). These results demonstrate that loss of CARM1 in GSCs not only results in increased NGFR/NTRK signaling but also increases dependency of GSCs on NGFR/NTRK signaling for survival.

### CARM1-dependent methylation of NFIA R389 contributes to repression of NGFR expression in glioma stem-like cells

CARM1, like other type I PRMTs, exerts its biological effects through placing ADMA on proteins and, loss of ADMA has repeatedly been shown to lead to a reduction in direct protein function and/or protein-protein/protein-nucleic acid interactions.^52–56^ To understand mechanistically how CARM1 may be regulating NGFR signaling, we used an antibody and mass spectrometry-based approach to define the known CARM1 substrates in NSC11 GSCs—a method previously used to identify CARM1 substrates in human breast cancer cells.^57^ Using an ADMA motif antibody, we isolated peptides with ADMA in both wild-type (WT) and CARM1-depleted cells and performed LC-MS to identify CARM1-specific substrates (Figure 5A). We compiled a list of known CARM1 substrates/interactors from various publications and compared our identified substrates to the available datasets. We found a total of 107 CARM1 substrates in our NSC11 GSC line, and all but 2 of those substrates have been shown to be arginine methylated previously^58^ (Table S4). Of our 107 substrates, we found 56 new human CARM1 substrates (Figure 5B) not identified in previous human studies on CARM1 substrates.^57,59^ To develop an understanding of the functions of the identified CARM1 substrates, we performed GO over representation analysis (ORA) to identify the molecular functions. The CARM1 substrates were almost all primarily involved in the regulation of transcription and DNA binding (Figure 5C; Table S4). We find that arginine 389 on NFIA (nuclear factor I A) is one of the most enriched CARM1 substrates in our cell line (Figure 5D).

NFIA is part of the family of NFI transcription factors that are known to regulate neural development,^60,61^ and have been confirmed to be CARM1 substrates in mouse embryonic fibroblasts.^62^ Moreover, using (chromatin immunoprecipitation sequencing) ChIP-seq, it has previously been shown that NGFR is a target of NFIA.^63^ Since NFIA specifically has been shown to be significantly involved in glioma tumorigenesis,^64–67^ we hypothesized NFIA to be the primary CARM1 substrate responsible for regulating NGFR signaling in GSCs. To test if NFIA is able to regulate NGFR expression in GSCs, we first used (small interfering RNA) siRNA to knockdown NFIA and probed NGFR expression with western blot. We found that knockdown of NFIA resulted in an upregulation of NGFR expression (Figure 5E), analogous to CARM1 depletion (Figure 5F). Since our results show that CARM1 methylates R389 on NFIA, we hypothesized that R389 methylation contributes to CARM1-dependent repression of NGFR in GSCs. To test this, we generated plasmids containing FLAG-tagged NFIA WT, NFIA R389A, or NFIA R389K and used a lentiviral approach to transduce the construct into NSC11 GSCs and validated the construct expression with western blotting (Figures 5G and 5H). Notably, NFIA R389A was used to alter both amino acid size and charge to disrupt methylation by any enzyme (including CARM1), and NFIA R389K was used to maintain size and charge but disrupt PRMT-specific methylation—a strategy consistently used in PRMT studies.^68–71^ Indeed, western blot analysis demonstrated that even without removing the endogenous NFIA, expression of mutant NFIA R389A or NFIA R389K increased NGFR expression similarly to that observed following CARM1 or NFIA depletion (Figure 5I). Since R389 is on the transactivation domain of NFIA—which interacts with transcriptional binding partners and not the DNA-binding region, we suspect that NFIA R389 methylation is likely involved in NFIA’s ability to recruit other transcriptional activators/repressors (Figure 5J). Overall, these results support a model in which CARM1-dependent methylation of NFIA R389 contributes to repression of NGFR signaling in GSCs.

### Loss of CARM1 suppresses GSC *in vivo* tumorigenicity

To further understand the role of CARM1 in regulating tumorigenicity of GSCs, we devised intracranial orthotopic xenograft studies. NSC11 sgSCR (*n* = 11) and sgCARM1-2 (*n* = 10) GSCs were implanted into NOD-SCID gamma (NSG) mice. Following implantation, microCT/positron emission tomography (PET) imaging was performed 30 and 60 days post-implantation, and mice in both groups were tracked until the end of the study (Figure 6A). NSC11 sgCARM1-2 GSCs had a significantly increased probability of survival compared to sgSCR cells; sgCARM1-2 GSCs had a median survival of 64 days compared to a median survival of 43 days for sgSCR cells—approximately a 49% increase in mouse survival in sgCARM1 tumors vs. sgSCR (Figure 6B). MicroCT/PET imaging at 30 and 60 days post-implantation demonstrated reduced *in vivo* tumor burden in sgCARM1 xenografts, whereas no mice bearing sgSCR control tumors survived to day 60 (last control mouse reached endpoint at day 49) (Figures 6C and 6D). Histological evaluation of orthotopic xenografts was further supported by SOX2/SOX9 immunofluorescence staining, confirming the presence of glioma stem-like tumor cells (Figures 6E and 6F)—known markers of glioma stemness and aggression.^72–75^ Notably, sgSCR GSCs demonstrated a more invasive nature with the majority of tumor cells found throughout the mouse corpus callosum in comparison to sgCARM1 GSCs, suggesting that CARM1 depletion may also alter glioma invasion patterns (Figures 6E and 6F). Ultimately, these findings demonstrate that depletion of CARM1 disrupts GSC *in vivo* tumorigenicity and prolongs mouse survival.

## DISCUSSION

Our work tests whether CARM1 is a candidate therapeutic target for GBM and how CARM1 regulates GSC character and developmental processes. We find that CARM1 positively regulates GSC cell proliferation and limits apoptosis, and its mRNA expression is increased in high-grade gliomas compared to low-grade gliomas. We also find that CARM1 has a substantial impact on the expression of developmental and chromatin-organizing genes and regulates the histone PTM profile of GSCs beyond the known H3R17 methylation. Proteomics and RNA-seq show that CARM1 restrains a shift toward a radial glial-like lineage state. Phospho-proteomics indicate that CARM1 depletion upregulates NGFR/NTRK signaling. In addition, CARM1-depleted GSCs show increased sensitivity to NTRK and AKT inhibitors. Mechanistically, mass spectrometry identifies arginine 389 on NFIA as a regulated CARM1 methylation substrate in GSCs, and mutation of NFIA arginine 389 to lysine or alanine increases NGFR expression, similar to CARM1 or NFIA depletion. These findings support a model in which CARM1-dependent methylation of NFIA contributes to repression of NGFR signaling in GSCs.

Though CARM1 has been investigated largely for its role in normal tissue development, less focus has been placed on its use as a therapeutic target in disease. One issue with targeting PRMTs is that there is redundancy in their substrates, and so when one PRMT is inhibited, others can compensate. This is not the case for CARM1. CARM1 methylates proline-arginine-rich motifs, while other PRMTs methylate glycine-arginine-rich motifs, thus CARM1’s differences in motif preference make its loss difficult to compensate.^14,76^ Moreover, CARM1 depletion has significantly less toxicity *in vivo* compared to depletion of other PRMTs (i.e., PRMT1 or PRMT5) and thus may be more tolerable in the clinic.^14,77,78^ Our work demonstrates that loss of CARM1 causes a reduction in GSC cell proliferation, increases apoptosis, and slows *in vivo* tumor progression—giving support to the proposal that CARM1 should be further investigated as a therapeutic target in GBM.

CARM1 has been previously reported to affect chromatin organization through its methylation of H3R17 and/or H3R26, and our data show that CARM1 depletion is associated with changes in several other histone PTMs. We observe that CARM1 depletion leads to an upregulation of H4K20me3 and bivalent H3K27me3K36me2, coupled with a downregulation of H3K18ac and H3K79me2. Likewise, we found differential expression of key enzymes that regulate these histone PTMs (i.e., PHF8, KDM6B, KDM2B, and KAT2A). Thus, CARM1 affects multiple histone PTMs in this system, likely through transcriptional control of histone PTM readers, writers, and erasers. These histone PTMs have been shown to play various roles in cellular development. H4K20me3 is often found on heterochromatin and associated with cell lineage commitment in embryonic stem cells.^79^ H3K27me3K36me2 is a bivalent histone mark that has been observed to be enriched in mesenchymal stem cells.^80^ Though this mark includes H3K36me3, our work has previously shown via MS- based histone analysis and ChIP/RNA-seq that bivalent H3K27me3K36me2 has a dominant repressive effect on transcription and chromatin accessibility.^81^ H3K18ac and H3K79me2 are histone PTMs often found at the TSS of genes undergoing active transcription and have meaningful roles in cellular development.^36,82,83^ The shifts in the histone PTM architecture upon CARM1 depletion are evident in a changing chromatin landscape in which there is a gain of repressive/heterochromatin marks and a loss of transcriptional activation/euchromatin marks. This is in line with the work of Mark Bedford’s group showing that CARM1 can promote an open chromatin state in small-cell lung cancer models.^62^ If indeed CARM1 depletion leads to a reduction in chromatin accessibility via histone PTM remodeling, this may have strong implications on the stem-like nature of GSCs, their lineage potential, and therapy resistance.

At both the transcript and protein levels, we observe upregulation of developmental markers such as RTN1 and GFAP, and downregulation of markers such as NOTCH1 and PDGFRA. These changes suggest that CARM1 loss does not necessarily drive terminal differentiation in GSCs, but instead shifts stem-like lineage programs toward a radial glial-like state. Radial glial-like cells have been identified in gliomas and can contribute to intra-tumoral heterogeneity,^41^ although their relationship to patient outcome remains incompletely defined. These findings support a role for CARM1 in regulating transcriptomic and proteomic features associated with radial glial-like programs in GSCs and contribute to understanding GSC plasticity.

One of the most upregulated genes/proteins upon CARM1 depletion in GSCs was NGFR. NGFR is a transmembrane receptor that stimulates the activity of NTRK1/2/3 and is vital to cell migration during neurodevelopment and tumorigenesis.^44–46^ Moreover, phospho-proteomics and western blot showed that sgCARM1 cells had upregulation of the entire NGFR/NTRK pathway. NTRK signaling plays a vital role in promoting the regeneration and survival of radial glial cells,^47^ consistent with our results showing that CARM1-depleted GSCs shift their lineage toward radial glial-like programs. The increased dependency on NGFR/NTRK signaling upon CARM1 loss can be exploited *in vitro* with direct inhibition of NTRK via entrectinib or through downstream inhibition of AKT kinase with capivasertib. These results highlight the value of defining chromatin-associated lineage regulators in GSCs, as they may uncover context-dependent therapeutic vulnerabilities in GBM. Although single-cell technologies can identify lineage-specific alterations in GBM, these studies often do not validate the dependencies observed in each lineage or the regulators that control those states. Defining which genetic and chromatin-associated regulators allow GSCs to switch between neural lineages may help refine therapeutic strategies and better predict recurrence. Further, to increase clinical relevance, additional studies should target distinct cell lineages within GBM using chemical or CRISPR-Cas9 screens to distinguish pathways that are differentially expressed from those required for survival.

NFIA is a CARM1 substrate,^62^ and in our dataset, it is one of the most differentially methylated CARM1 substrates. Loss of protein arginine methylation can disrupt protein function and cellular processes,^53,84–86^ and the pronounced decrease in NFIA R389 methylation (log_2_FC < −7 in CARM1-depleted vs. WT) suggests that this modification may affect NFIA transcriptional function. Because the NGFR promoter is bound by NFIA,^63^ NFIA is a plausible CARM1 substrate involved in regulating NGFR. NFIA knockdown recapitulates the increased NGFR expression observed after CARM1 depletion. Mutational studies further show that substitution of NFIA R389 with lysine or alanine increases NGFR expression, similarly to depletion of CARM1 or NFIA. Additional studies will be needed to define how loss of methylation at NFIA R389 affects NFIA function. Based on AlphaFold predictions, R389 is not located within the DNA-binding region of NFIA, suggesting that loss of methylation may affect NFIA protein interactions rather than DNA binding. It is possible that loss of R389 methylation disrupts NFIA interactions with co-repressors, allowing NGFR expression to increase.

### Limitations of the study

This study defines a role for CARM1 in regulating GSC developmental programs, NGFR/NTRK signaling, and orthotopic tumorigenicity, but several limitations remain. The functional analyses include growth, apoptosis, clonogenic survival, drug response, and *in vivo* tumor formation, but do not include limiting-dilution neurosphere assays, serial sphere formation, or serial transplantation; therefore, conclusions regarding GSC self-renewal should remain limited. Most mechanistic experiments emphasize NSC11 cells, and additional patient-derived models will be needed to determine how broadly the CARM1-NFIA-NGFR axis applies across GBM subtypes. Finally, the therapeutic implications of increased NTRK/AKT inhibitor sensitivity after CARM1 depletion were tested *in vitro*, and future *in vivo* treatment studies will be required to evaluate whether this dependency can be exploited therapeutically.

These results indicate that CARM1 regulates gene and protein expression of key developmental markers in GSCs and restrains a shift toward a radial glial-like cell state. NGFR/NTRK signaling, a pathway involved in radial glial cell survival, is upregulated upon CARM1 loss and supports GSC survival *in vitro*. The increase in NGFR/NTRK signaling upon CARM1 loss is consistent with a model in which CARM1-dependent methylation of NFIA R389 contributes to NGFR repression. Finally, CARM1 loss reduces GSC *in vivo* tumorigenicity and increases mouse survival. Overall, these findings support a model in which CARM1 helps maintain GSC lineage state and survival signaling programs that may be therapeutically targetable.

## RESOURCE AVAILABILITY

### Lead contact

Further information and requests for resources and reagents should be directed to and will be fulfilled by the lead contact, Simone Sidoli (simone.sidoli@einsteinmed.edu).

### Materials availability

Plasmids generated in this study are available from the lead contact upon request. This study did not generate new mouse lines.

### Data and code availability

- The mass spectrometry data have been deposited to the ProteomeXchange Consortium via the PRIDE^87^ partner repository with the dataset identifier PXD080227. Raw sequencing data have been deposited in the NCBI Sequence Read Archive under BioProject accession no. PRJNA1237514. Processed gene expression data have been deposited in the NCBI Gene Expression Omnibus database under accession no. GSE292789.
- This paper does not report original code.
- Any additional information required to reanalyze the data reported in this paper is available from the lead contact upon request.

## STAR★METHODS

### EXPERIMENTAL MODEL AND STUDY PARTICIPANT DETAILS

#### Cell culture

Glioma stem-like cell lines (GSCs) NSC11 and NSC20 are two patient derived cell lines provided as frozen stocks by Dr. Frederick Lang, MD Anderson Cancer Center, Houston, TX. GSCs were grown as neurospheres in stem cell media (DMEM/F12, 50ng/mL EGF and bFGF, 2% B27 without vitamin A, and 1% penicillin-streptomycin) and GSCs were characterized as previously reported.^88^ All cell lines were maintained in culture (5% O_2_ and 5% CO_2_ incubators) for less than two months from thawing and confirmed to be negative for mycoplasma with PCR. GSCs cell morphology and growth was constantly monitored for health, and NSC11 and NSC20 cell line differences were authenticated using proteomics.

NSC11 and NSC20 are human patient-derived models; no participants or fresh human biospecimens were prospectively enrolled or collected for this study. Donor age, sex, gender, ancestry, race, and ethnicity were not available for these established lines, and associations with these variables could therefore not be evaluated. The two lines represent independent patient-derived models. For genetic perturbation experiments, cells were allocated to sgSCR, sgCARM1-1, or sgCARM1-2 conditions by lentiviral transduction as described below. Line identity was assessed using distinct global proteomic profiles, and all GSC cultures tested negative for mycoplasma by PCR.

#### HEK293T packaging cells

Human HEK293T cells were used exclusively for lentiviral particle production by calcium phosphate transfection and were not included in biological comparisons. The source, donor sex, authentication method, mycoplasma-testing status, and detailed maintenance conditions for this packaging line were not recorded in the available study documentation.

#### Animals

All animal experiments were approved by the Institutional Animal Care and Use Committee at the Albert Einstein College of Medicine in accordance with the National Institutes of Health (NIH) guidelines (approved protocol no. 00001516). Eight- to ten-week-old NOD-SCID gamma (NSG) mice (NOD.Cg-Prkdcscid Il2rgtm1Wjl/SzJ) were purchased from The Jackson Laboratory (stock no. 005557) and used for all studies. Mice were housed under specific pathogen-free conditions in individually ventilated cages with controlled temperature and humidity (ScanClim, Scanbur, Denmark), maintained on a 12-h light/dark cycle, and provided food and water *ad libitum*. In the orthotopic xenograft study, 11 mice received NSC11 sgSCR cells and 10 mice received NSC11 sgCARM1-2 cells; experimental groups were defined by the implanted cell population. The sex of the mice was not recorded in the available study documentation; consequently, sex-associated effects could not be evaluated, which is a limitation of the study.

### METHOD DETAILS

#### Intracranial tumor implantation

Human Glioma stem-like cell (GSC) sphere cultures were dissociated into single cells prior to implantation. Cell viability was between 88 and 94%. A total of 2 × 10 ^5 cells suspended in 5 μL DMEM/F12 were stereotactically injected intracranially as previously described.^89^ Following surgery, mice were allowed to recover and were closely monitored for general health and neurological symptoms. Animals were euthanized upon onset of neurological signs in accordance with predefined humane endpoint criteria.

#### MicroCT/PET imaging

microCT/PET imaging was performed using an Inveon multimodality scanner (Siemens INVEON PET/SPECT/CT Trimodal System). For *in vivo* imaging, a custom-designed four-mouse nose cone/holder was built by Dr Koba to enable simultaneous imaging of four animals at once, as previously described.^90^ Mice (WT and KD) were anesthetized using isoflurane (1.5% in oxygen at a flow rate of 1.5 L/min) delivered via a VetEquip anesthesia system. Following induction, animals were positioned in the imaging holder and maintained under anesthesia for the duration of the scan (45min). After imaging, mice were allowed to recover in a warmed cage placed on a heating pad (temperature maintained below 26°C).

For CT acquisition, a step-and-shoot protocol was used with the following parameters: half rotation, 220 projections, 594 s scan time, low-resolution magnification, binning factor of 2, effective pixel size of 46.4 μm, 2496-pixel transaxial CCD (57.9 mm field of view), 4032-pixel axial CCD (93.53 mm field of view), 94 mm scan length, tube voltage of 80 kV, current of 500 μA, and a 1.5 mm filter. Exposure time was 925 ms per projection.

For PET imaging, overnight fasted mice were injected intraperitonially with [^18F]fluorodeoxyglucose (FDG) (270–350 μCi per mouse) and allowed an uptake period of approximately 40min under anesthetized conditions. Static PET acquisition was then performed for 12min. Data were acquired using a standard energy window of 350–650 keV and reconstructed using an ordered subset expectation maximization (OSEM) algorithm with attenuation correction based on CT data.

Raw imaging data were reconstructed, cropped, and exported as Digital Imaging and Communications in Medicine (DICOM) files using Inveon Acquisition Workplace software (Siemens). Image orientation was corrected using Inveon Research Workplace, and datasets were subsequently visualized and analyzed using Inveon Research Workplace (Siemens). Standardized uptake values (SUVs) were quantified in three anatomical regions: the tumor site in the right hemisphere (RH), the contralateral left hemisphere (LH), and the cerebellum (CB), using PET tissue windows and CT bone windows for anatomical reference.

#### Immunohistochemistry and microscopy

The dissected mouse brain tissue was fixed in 4% paraformaldehyde for 48 hours, and cryoprotected in 20% sucrose for another 24 hours, followed by 30% sucrose for another 24 hours, then snap frozen using liquid nitrogen. Twenty micrometer cryostat sections were prepared and kept at −20°C. For immunohistochemistry staining, frozen sections were allowed to be defrosted for 15 min, rehydrated 3 × 5 min in PBS, and then 2 × 5 minutes in BD perm buffer (5X) and incubated with directly conjugated antibodies diluted in blocking buffer (5X perm buffer+4% FBS). The SOX2-AF488 (eBioscience) and SOX9-AF488 (Novus) were used (1:400), incubated overnight at 4°C, then washed 2 × 5 min in PBS followed by DAPI staining for 5 minutes (1:10000) before being mounted with Prolong Gold anti-fade reagent and kept to dry for 24h before microscopy.

For microscopy, High-resolution images of histological sections were examined and acquired at 40× magnification using an automated AEON Cytoscanner and analyzed using AEON analytics Software (Coluxa Inc, Fremont, CA) and Figure platform made in Inscape.

#### Lentiviral CRISPR/Cas9

We used a two plasmid approach to ablate target genes. The lentiCas9-Blast (plasmid #52962) and pXPR_050 (plasmid #96925) was purchased from Addgene. Custom sgRNA sequences were made with CRISPick and cloned as described previously.^91^ Lentiviral particles were produced in HEK293T cells by calcium phosphate transfection. Lentivirus was next concentrated using polyethylene glycol (Systems Biosciences LV810A-1) and stored in cryotubes in −80°C. Transduction of GSCs was performed by mixing 25 μl of concentrated lentivirus with 1 × 10^6^ GSCs in 100 μl of stem cell media and incubated for 2 h at 5% O_2_ and 5% CO_2_. After lentiviral infection GSCs were cultured for 5 days in normal stem cell media, then selected for 8 days with 10 μg/mL of blasticidin (ThermoFisher A1113903) and/or 4 μg/mL of puromycin (Sigma P9620). Gene depletion was then validated with western blot. Cells were utilized for experiments for no longer than a month.

sgCARM1-1: sense: 5′-AGCACGGAAAATCTACGCGG-3′
antisense: 3′-CCGCGTAGATTTTCCGTGCT-5′
sgCARM1-2: sense: 5′-TGGAGCACGGAAAATCTACG-3′
antisense: 3′-CGTAGATTTTCCGTGCTCCA-5′

#### siRNA knockdown

We used lipofectamine RNAiMAX transfection reagent (ThermoFisher) to generate siRNA knockdowns. We purchased two different siRNA against NFIA (ThermoFisher s9477, s9478) and one against NGFR (ThermoFisher s9540). We followed the RNAiMAX protocol for transfection. In short, 1 × 10^6^ GSCs were seeded into 10mL of stem cell media with 40nM of siRNA-lipofectamine complexes against siRNA of target gene or siCTRL (negative control) and placed in incubator for 24 h. GSCs were then gently collected, spun down, and reseeded with fresh media without siRNA and placed in incubator for another 4 days. Fresh media was added accordingly. siRNA sequences are below:

siNFIA-1: sense: 5′-CGAAAACGAAAAUACUUCAtt-3′
antisense: 5′-UGAAGUAUUUUCGUUUUCGgg-3′
siNFIA-2: sense: 5′-CACAUCACCCAUUAUCCAtt-3′
antisense: 5′-UGGAUAAUGGGUGAUGUCGgg-3′
siNGFR: sense: 5′-CCACAGCAGGUGUCAUAUAtt-3′
antisense: 5′-UAUAUGACACCUCUGUGGtg-3′

#### Western blot

Fifteen to twenty micrograms (lysed with RIPA buffer and measured with BCA, ThermoFisher) of cell lysates were mixed with 2–4x Laemmli Sample Buffer and β-mercaptoethanol, then ran down SDS-PAGE 4–15% gradient gel and transferred to a PVDF membrane. The membrane was blocked with 5% non-fat dry milk in Tris-Buffered Saline (TBS)-Tween 0.1% for 1 h. The membrane was then incubated with blocking buffer and various antibodies: CARM1 (ThermoFisher A300-421A 1:2000), NGFR (Cell Signaling #8238 1:1000), ERK1/2 (Cell Signaling #9102 1:1000), phospho-ERK1/2 (Cell Signaling #4370 1:1000), NFIA (Cell Signaling #69375 1:1000), AKT (Cell Signaling #4691 1:1000), phospho-AKT (Cell Signaling #9271 1:1000), GAPDH (Santa Cruz #47724 1:1000).

#### Cell viability and drug treatments

For growth curves, 1.5 × 10^3^ GSCs were seeded in 100 μl of stem cell media in opaque 96 well plates, growth was measured on day 0, 3, and 6 with Cell Titer-Glo 2.0 (Promega G9242) and Student’s *t* test was applied for statistical significance. For drug treatments, 5 × 10^3^ GSCs were seeded into 100 μl of stem cell media with various dosages of either Entrectinib (MedChemExpress HY-12678) or Capivasertib (MedChemExpress HY-15431) for 96 h and compared to DMSO vehicle controls. Cell viability was then measured with Cell Titer-Glo 2.0. Drug curves were plotted with Prism (version 10) non-linear regression best fit curve for visualization, data is reported as mean ± SEM.

#### Clonogenic assays

For clonogenic assays,^92^ GSCs were dissociated into single cells and 100–500 cells were seeded onto poly-L-lysine (sigma) coated 6 well plates in stem cell media and allowed to adhere overnight. Media was then removed and replaced with media containing 3 μM of Entrectinib or DMSO and incubated for 72 h. Media was then replaced with drug free media and colonies were allowed to form for 16 days. Next, colonies were stained and fixed with 0.5% crystal violet in 10% neutral buffered formalin. The number of colonies containing ≥50 cells were counted for sgSCR, sgCARM1-1, and sgCARM1-2. Entrectinib colony formation efficiency was normalized to DMSO colony formation efficiency for each sgRNA to account for differences in plating efficiencies. Data is reported as mean ± standard deviation. The Student’s *t* test was performed for statistical evaluation with alpha level of 0.05 for statistically significant results.

#### Apoptosis assay

Apoptosis was assessed using an AnnexinV/PI staining assay followed by flow cytometry. GSCs spheroids were collected, washed with PBS and TryplE were added. After 3 min incubation at 37°C the cells were washed two times with PBS. The cells were resuspended in 1x AnnexinV binding buffer prepared from 10X stock (BD Pharmingen, 51-66121E). A total of 100 μl of cell suspension was incubated with 5 μl FITC-AnnexinV (BD Pharmingen, 556419) and 5 μl PI (EMD Millipore, 537060) per sample. Samples were gently mixed and incubated for 15 min at room temperature in the dark. Thereafter, 400 μl of 1x AnnexinV binding buffer was added to each sample, and cells were immediately analyzed by flow cytometry. The flow cytometry data were analyzed using FlowJo software.

#### Cell cycle analysis

GSC spheroids were collected, washed with PBS and TrypleE were added. After 3 min incubation at 37°C the cells were washed two times with PBS. Cells were fixed in 70% ethanol and stored at −20°C overnight. Fixed cells were washed twice with PBS and resuspended in staining solution containing 1 μg/mL DAPI (Invitrogen, Cat# D3571) and 100 μg/mL RNase A (EMD Millipore, Cat# 70856). Cells were incubated at 37°C for 30 min and subsequently analyzed by flow cytometry. Flow cytometry data were processed using FlowJo software, and quantitative analysis was done using GraphPad Prism.

#### Differentiation analysis

GSCs were dissociated into single cells, and 2 × 10^6^ cells were seeded into T75 flasks containing differentiation media (DMEM high glucose with glutamine, 1% pen/strep, 1mM sodium pyruvate, and 10% FBS). Control cells were maintained in stem cell media. Media was replaced every 48 h and cells were collected after 7 days and used for proteomics.

#### Generation of NFIA arginine mutant

In order to test the function of NFIA R389 ADMA, lentiviral based plasmids overexpressing flag-tagged WT NFIA or NFIA R389A/K mutations were created and transduced into GSCs. First, NFIA R389 perturbations were generated by creating NFIA WT or synonymous mutations of NFIA R389A/K using Geneious Prime. To generate synonymous mutations for NFIA, bases between 1165 and 1167 were changed to ‘GCC’ to create NFIA R389A and bases 1165–1167 were changed to ‘AAG’ to create NFIA R389K. In the event that expression of the mutant NFIA was not sufficient to investigate our phenotype of interest due to the presence of endogenous NFIA, we designed a silent mutational strategy in which our exogenous NFIA WT, NFIA R389A, and NFIA R389K would be resistant to our siNFIA-1 sequence (see siRNA knockdown section for siNFIA-1 sequence). NFIA WT, NFIA R389A, NFIA R389K constructs all included the sequence ‘CGGAAGCGGAAGTATTTTA’ between bases 106–124 so they could not be targeted by siNFIA-1, if necessary. Using the VectorBuilder website, NFIA WT, NFIA R389A, NFIA R389K were constructed to be placed under an EF1A promoter and the lentiviral based plasmid included a puromycin resistance gene and were purchased. The complete vector sequences are available in Table S5.

GSC transduction followed the same protocol as mentioned previously. In short, transduction of GSCs was performed by mixing 25 μl of concentrated lentivirus with 1 × 10^6^ GSCs in 100 μl of stem cell media and incubated for 2 h at 5% O_2_ and 5% CO_2_. After lentiviral infection GSCs were cultured for 5 days in normal stem cell media, then selected for 8 days with 4 μg/mL of puromycin (Sigma P9620). Presence of exogenous NFIA was then validated with western blot of FLAG tag.

#### Proteomics sample preparation

Cell pellets were prepped and digested based on S-trap proteomic filters (Protifi) based on the manufacturer’s instructions. Briefly, dry cell pellets were resuspended with 50 μl of 5% SDS and 1 μl of Pierce universal nuclease (ThermoFisher 88701), followed by 1 h incubation in DTT (dithiothreitol, Sigma) at 37°C, and a 30 min incubation in 20mM iodoacetamide (Sigma). Next, phosphoric acid was to samples at a concentration of 1.2% and proteins were diluted in S-trap binding buffer (90% methanol and 10mM ammonium bicarbonate), then added to S-trap filters. Samples were spun down for 1 min at 500G, then filters were washed three times with S-trap binding buffer. One 1 μg of sequence grade trypsin (Promega) or chymotrypsin (Promega) was mixed with 50mM ammonium bicarbonate and added to samples in S-trap filters. Proteins were then digested overnight at 37°C, then sequentially eluted with 60 μl of 50mM ammonium bicarbonate, 0.1% trifluoroacetic acid (TFA), and 60% acetonitrile/0.1% TFA. Peptides were then completely dried in a speed vacuum and stored in −80°C freezer until desalting or other purposes such as CARM1 substrate identification or phospho-proteomics.

For identification of CARM1 asymmetric di-methyl arginine (ADMA) substrates we used the PTMScan kit (Cell Signaling #18303S) as previously,^57^ and largely followed the kit instructions. Briefly, after drying of S-trap eluted peptides as described above, peptides were resuspended in PTMScan IAP buffer (10% of samples were saved for analysis of total proteome, 90% used for PTMScan) and mixed with appropriate volume of ADMA antibodies bound to magnetic beads and rotated overnight at 4°C. After several washes peptides were eluted off magnetic beads with IAP Elution Buffer (0.15% TFA +99.85% water), dried in speed vacuum, and stored in 80°C freezer.

For identification of phospho-peptides we used a previous protocol as described here.^93^ In short, after elution and drying of digested peptides, peptides were resuspended in 100 μl of 0.1% TFA and 10% of sample was taken for total proteome analysis, remaining 90% proceeded with phosphorylation pulldown. We added 0.6 mg of titanium beads (GL Sciences #5010–21315) per 100 μg of protein and incubated for 10 min with shaking at 1400 rpm, removed supernatant carefully. We repeated this process one more time to capture all phospho-peptides. After several washes phospho-peptides were eluted with Elution buffer (80μl of Ammonium Hydroxide +980 μl HPLC water, pH = 11.3), dried in speed vacuum, and stored in −80°C freezer.

#### Histone sample preparation

Histones were extracted as previously described.^35,94^ Dry cell pellets were dissolved in 350 μl of 0.2 M sulfuric acid and incubated for 4 h at 4°C with rotation. Samples were mixed with 150 μl (or 33%) trichloroacetic acid (TCA) overnight at 4°C. Then samples were spun down for 3400G for 5 min, and the supernatant was then disposed of. Next histones were sequentially washed with 0.1 HCL in cold acetone and 100% cold acetone, then air dried. Histones were then resuspended in 50 μl of 50mM ammonium bicarbonate with 20% acetonitrile. In the fumigation hood, histones were derivatized with addition of 2 μl of propionic anhydride and 10 μl of ammonium hydroxide were added and incubated for 10 min, these steps were then repeated once again and samples were then dried in a speed vacuum. Next, 1 μg of sequencing grade trypsin was diluted in 50mM Ammonium Bicarbonate and incubated overnight at 37°C then derivatized again the following day. Samples were then dried in a speed vacuum and stored in −80C freezer.

#### Sample desalting

Before mass spectrometry analysis samples were desalted with 1mg of Oasis HLB C-18 resin (Waters) in 96-well filter plates (Orochem). Briefly, plates were loaded with HLB C-18 resin, then washed, and samples resuspended in 0.1% TFA were added to plates. Samples were washed with 0.1% TFA, then eluted with 0.1% TFA/60% Acetonitrile, and dried in a speed vacuum.

#### Gene set enrichment analysis

Gene Set Enrichment Analysis was performed using the R (v4.3.1) package clusterProfiler.^95^ Gene sets used were from MSigDB C2 collection and Gene Ontology. For proteomics data, we averaged the log2foldchange and −log_10_*p*-value of sgCARM1-1 and sgCARM1-2 then the data was rank-ordered by (sign (log2foldchange) * −log_10_*p*-value). For RNA-seq the data was also rank-ordered by (sign (log2foldchange) * −log_10_adjp-value).

#### TCGA analysis

To access TCGA data of CARM1 expression in lower grade glioma and GBM patients we used UCSC Xena browser.^96^ Data analysis was performed with R (v.4.3.1) with survival and survminer libraries.

#### Proteomic analysis

After desalting, samples were resuspended in 0.1% Formic Acid (FA) and loaded onto the Bruker nanoElute 2 coupled online with the timsTOF HT (Bruker). Chromatography separation was used using a two-column system consisting of a C18 trap cartridge (ThermoFisher #174500) and an analytical PepSep XTREME column of 25 cm × 150 μm ×1.5 μm (Bruker #1893476). Samples were separated using a 45 min gradient from 4 to 30% buffer B at a flow rate of 700nL (Buffer A: 0.1% FA, Buffer B: 80% acetonitrile +0.1% FA). The spectra were acquired in dia-PASEF in the range of 100–1700 m/z and 0.6 1/K0 [V-s/cm-2] to 1.6 1/K0 [V-s/cm-2]. Ramp and accumulation times were set to 100. 32 DIA isolation windows were used in the range of 400–1201 m/z and 0.6 1/K0 [V-s/cm-2] to 1.6 1/K0 [V-s/cm-2]. The collision energy was ramped as a function of increasing mobility starting from 20 eV at 0.60 1/K0 to 59 eV at 1.6 1/K0. The MS raw data was searched using FragPipe version 22^97^ with the preloaded workflow “DIA_ SpecLib_Quant_diaPASEF”. Our digestive enzymes were trypsin or chymotrypsin (for CARM1 substrate analysis only). Using R, raw protein abundances were log2 transformed, normalized by median centering, and imputed using a normal distribution two standard deviations beneath the mean as described previously.^87^ For CARM1 ADMA substrate and phospho-proteomics analysis we added another normalization step in which normalized ADMA peptides and phospho-peptides were divided by the normalized total protein abundance of their respective proteins. This approach allows us to quantify the differential abundance of the specific PTMs while removing any bias that may be introduced due to differences in total protein abundance. For statistical analysis, equality of variances between groups was first assessed using an F-test, followed by either Welch’s *t* test for unequal variances or Student’s *t* test for equal variances with alpha level of 0.05 for statistically significant results. Proteins/peptides were considered differentially expressed only if they were statistically significant in both sgCARM1-1 and sgCARM1-2.

#### Histone post-translational modification (PTMs) analysis

Post desalting, samples were resuspended in 0.1% TFA and placed onto the Dionex RSLC Ultimate 3000 and analyzed by an Orbitrap Fusion Lumos (ThermoFisher). A two column system was used to separate the histone peptides composed of a C18 trap cartridge (300 μm ID, 5 mm length) and an analytical column (75 μm, 25 cm). The analytical column was packed in our lab with reversed-phase Repro-Sil Pur C18-AQ 3 μm resin. Histone peptides were separated using a 30 min gradient and at a flow rate of 300nL/min in DIA mode. The full MS scan was set to 300–1100 m/z in the orbitrap with a resolution of 120,000 (at 200m/z).

Raw data files were searched through EpiProfile 2.0 software,^98^ and the area under the curve was generated for each identified histone peptide. To properly normalize the data, the sum of every identified combination for a histone peptide was summed (total peptide abundance) and considered to be 100% and the area of each individual peptide was divided by the total peptide abundance to generate a ratio. The relative ratio of two isobaric forms was calculated by averaging the ratio for each fragment ion between the two. Using R, the final peptide table was log2 transformed, log2foldchange was calculated, and statistical testing was performed by first assessing equality of variances between groups using an F-test, followed by either Welch’s *t* test for unequal variances or Student’s *t* test for equal variances with alpha level of 0.05 for statistically significant results. All histone PTMs were only considered differentially expressed if they were statistically significant in both sgCARM1-1 and sgCARM1-2.

#### Bulk mRNA transcriptome differential expression analysis

RNA was extracted from cell pellets using the RNeasy kit (Qiagen), quantified using a Qubit kit (Thermo Fisher Scientific) and high purity was confirmed by 260/280 and 260/230 ratios using a Nanodrop (Thermo Fisher Scientific). Libraries were prepared for mRNA analysis and sequenced on an Illumina Novaseq X using 150bp paired-end mode by Novogene Corp Inc., CA. Raw sequence reads were processed using the nf-core rnaseq pipeline (https://nf-co.re/rnaseq/3.14.0) using the star_rsem alignment option and min_mapped_reads set to 5 to align the reads to the GRCh38 primary genome assembly and gene level counts were obtained using the gencode v42 primary assembly annotation.^99^ Gene filtering was performed using the zFPKM program.^100^ Correlation analysis of TPM values between replicate samples was performed to ensure high concordance. Since there were no significant differences between sgCARM1-1 and sgCARM1-2 we combined these replicates and compared them to sgSCR. Count normalization and differential expression testing was performed using DESeq2 and genes were considered significantly enriched if the adjusted *p*-value <0.05.

#### Qiagen Ingenuity Pathway Analysis (IPA) and over representation analysis

For analysis of only differentially expressed genes we used two tools, Gene Ontology over representation analysis (ORA) and Qiagen IPA. To identify the Gene Ontology molecular functions of CARM1 substrates we used clusterProfiler in R. We used Benjamini-Hochberg for multiple testing correction, only considered GO terms with an adjusted *p*-value <0.05, and used all the identified proteins in the dataset as our background. For phospho-proteomic analysis we used Qiagen’s Ingenuity Pathway Analysis (IPA) software.^101^ We uploaded our table of differentially expressed phospho-peptides including the phospho-sites to IPA and selected the default phosphorylation analysis as our core analysis. We downloaded tables of most enriched canonical pathways and upstream regulators. For upstream regulators we filtered out chemical drug/reagents/toxicants as this was not relevant to our work and sorted by descending bias-corrected *Z* score, only including regulators with *p*-value <0.05. For canonical pathways, we filtered for pathways with a *p*-value <0.05 and an absolute *Z* score of 2 or greater, then sorted in descending order based on *Z* score. Qiagen IPA results were visually represented with dot plots from R using the ggplot2 library.

### QUANTIFICATION AND STATISTICAL ANALYSIS

Statistical analyses were performed using R (v4.3.1), GraphPad Prism (v10), FlowJo, FragPipe (v22), EpiProfile (v2.0), nf-core/rnaseq (v3.14.0), DESeq2, clusterProfiler, and QIAGEN Ingenuity Pathway Analysis, as appropriate; software details are listed in the key resources table. Exact sample sizes, the statistical test used for each experiment, and the definitions of error bars are reported in the corresponding figure legends. For cell-based assays, n denotes independent biological experiments; for xenograft studies, n denotes individual mice. Unless otherwise indicated, data are presented as mean ± SEM or mean ± SD, as specified in the figure legends. Growth, drug-response, and clonogenic-assay data were evaluated using Student’s t tests. Apoptosis data were analyzed by two-way ANOVA with Tukey’s multiple-comparisons test. For proteomic, phosphoproteomic, ADMA-enrichment, and histone-PTM analyses, equality of variance was first assessed using an F test, followed by Student’s *t* test for equal variances or Welch’s *t* test for unequal variances. Proteins, peptides, and histone PTMs were considered differential only when they met the stated significance threshold in both sgCARM1-1 and sgCARM1-2 comparisons. Raw protein abundances were log2-transformed, median-centered, and missing values were imputed from a normal distribution centered two standard deviations below the mean. ADMA-peptide and phosphopeptide abundances were additionally normalized to the corresponding total-protein abundance. Histone PTM abundance was expressed relative to the total abundance of all modified and unmodified forms of the corresponding peptide. RNA-seq counts were normalized and tested using DESeq2; genes with Benjamini-Hochberg-adjusted *p* < 0.05 were considered significant. Gene Set Enrichment Analysis was performed with clusterProfiler using the ranked statistics described above. Over-representation analyses used Benjamini-Hochberg correction and an adjusted *p* < 0.05 threshold. QIAGEN IPA canonical pathways were retained at *p* < 0.05 and |*Z* score| ≥ 2, and upstream regulators at *p* < 0.05. Kaplan-Meier survival curves were compared using the log rank (Mantel-Cox) test. A *p* value <0.05 was considered statistically significant unless otherwise stated.

## Supplementary Material

1

2

3

4

5

6

Supplemental information can be found online at https://doi.org/10.1016/j.celrep.2026.117785.

## Figures and Tables

**Figure 1.**
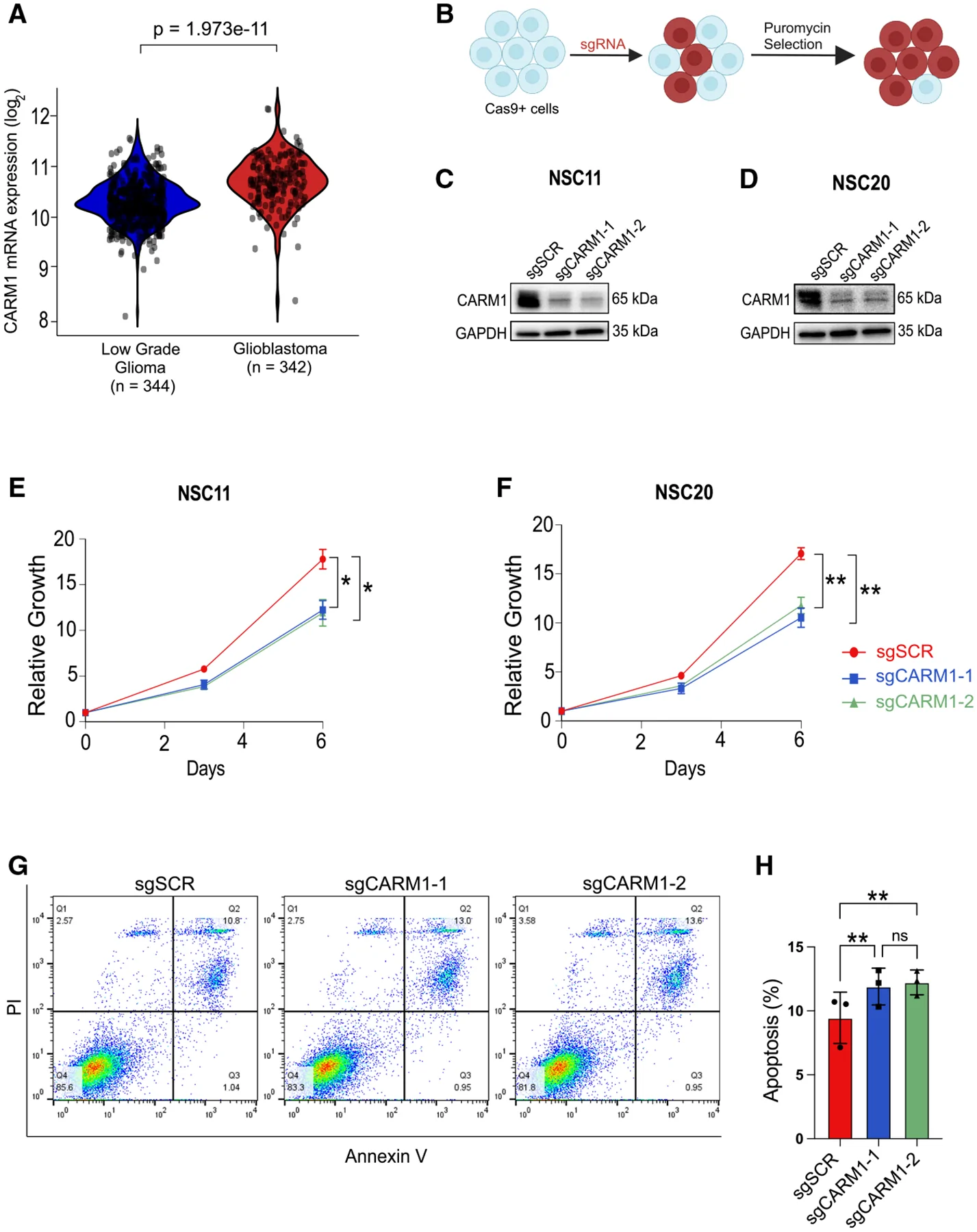
CARM1 predicts glioma patient survival and regulates glioma stem-like cell proliferation (A) Violin plot of mRNA expression log_2_ (normalized count+1) of CARM1 in low-grade glioma (*n* = 344) and glioblastoma (*n* = 342) using the UCSC Xena Browser. (B) Schematic CARM1 depletion approach. Cas9+ GSCs were generated using a lentivirus-based approach, followed by transduction of sgRNAs against either CARM1 or non-targeting controls (sgSCR). Negative selection with puromycin was used to isolate CARM1 depleted or control populations. (C and D) Western blot of CARM1 following CRISPR-Cas9 depletion and negative selection in NSC11 and NSC20 GSCs. (E and F) Growth curves of sgSCR and sgCARM1 ablated NSC11 and NSC20 cells measured with Cell Titer Glo 2.0, *n* = 3, data show mean ± SEM. Statistical analysis was performed with Student’s *t* test. (G and H) Representative flow cytometry density plots of Annexin V and propidium iodide staining in sgSCR, sgCARM1-1, and sgCARM1-2 NSC11 GSCs along with (H) quantification of apoptosis. Data represent mean ± SD from *n* = 3 independent experiments. Statistical significance was determined using a two-way ANOVA with Tukey’s test. ns, not significant; *p < 0.05; **p < 0.01; ***p < 0.001; ****p < 0.0001.

**Figure 2.**
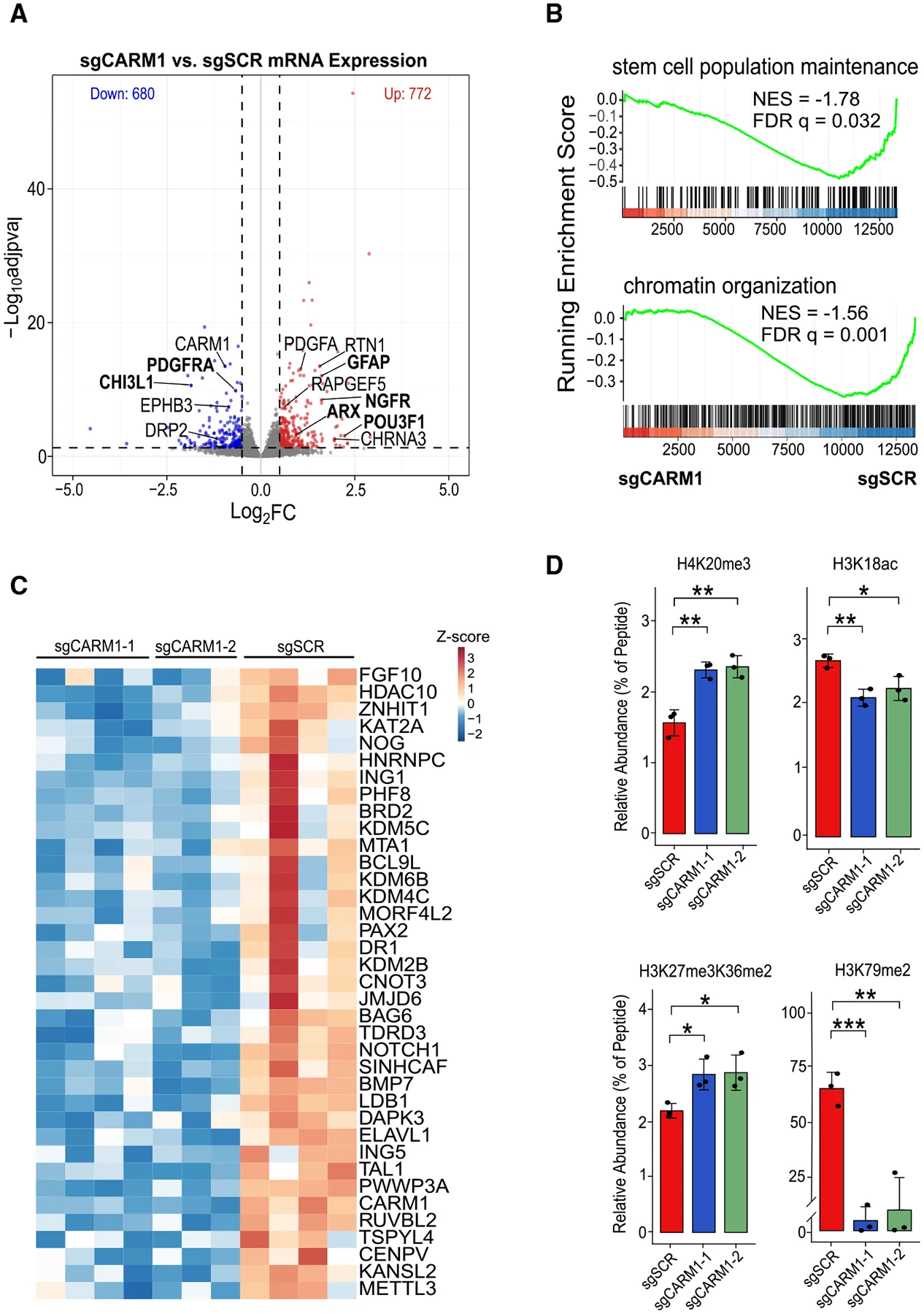
CARM1 regulates widescale transcriptome and histone PTMs involved in development (A) Volcano plot of NSC11 sgCARM1 (combined sgCARM1-1 and sgCARM1-2) vs. sgSCR highlighting key developmental genes (adjusted *p* value < 0.05 and n = 3–4 replicates per sgRNA). (B) GSEA Enrichment plots of RNA-sequencing data of NSC11 sgSCR and sgCARM1 cells for “stem cell population maintenance” (upper) and chromatin organization (lower) gene sets. (C) Heatmap of differentially expressed genes involved in chromatin organization (adjusted *p* value < 0.05) scaled by *Z* score of mRNA-normalized counts. (D) Mass spectrometry analysis of significant histone post-translational modifications (PTMs) in NSC11 sgSCR and sgCARM1 cells, data show mean ± SD. Statistical analysis was performed with Student’s or Welch’s *t* test *p < 0.05; **p < 0.01; ***p < 0.001; ****p < 0.0001.

**Figure 3.**
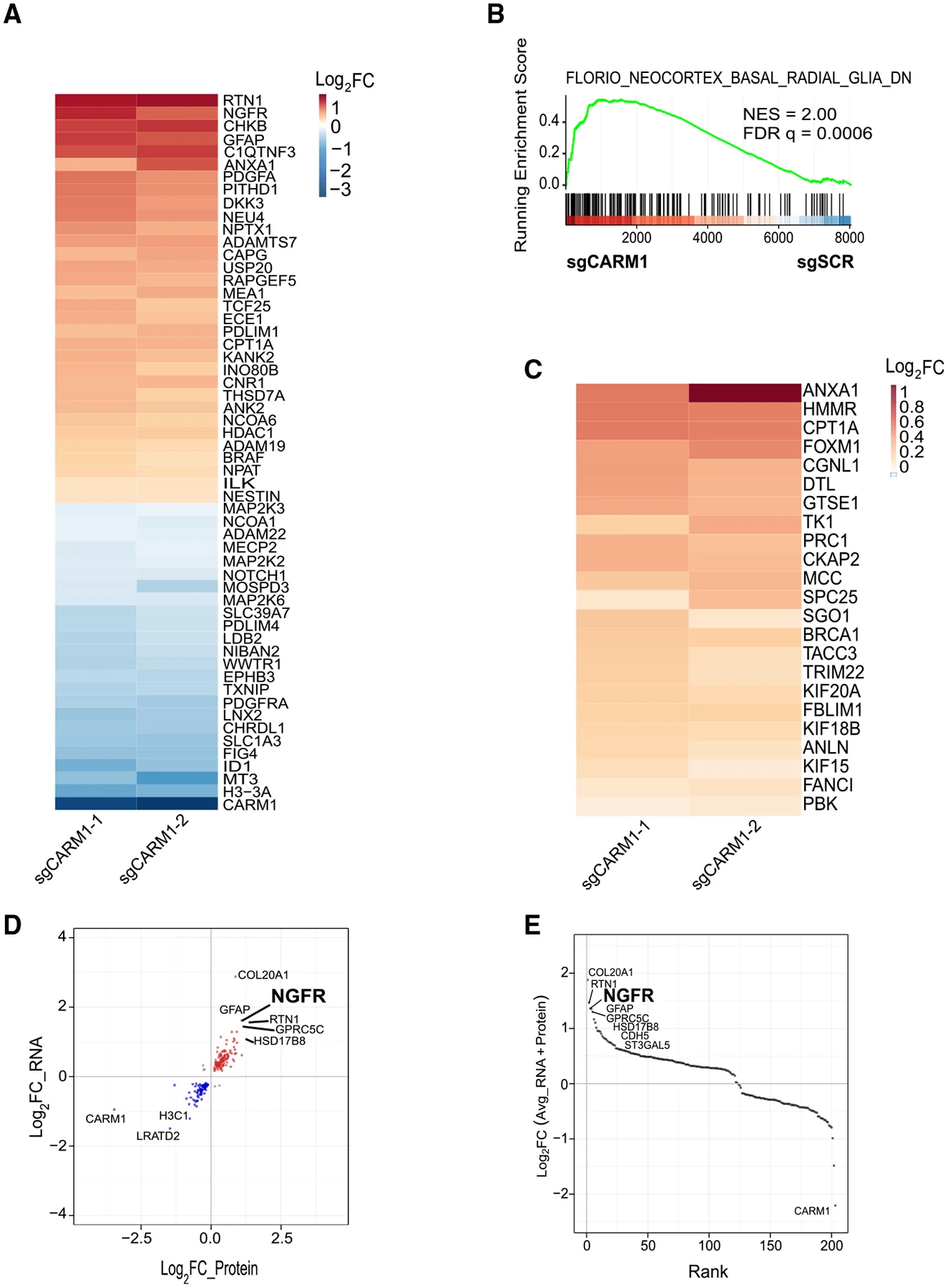
CARM1 represses radial glial cell proteomic signature in glioma stem-like cells (A) Proteomics-based heatmap of key differentially expressed proteins in NSC11 sgCARM1-1 and sgCARM1-2 (compared to sgSCR) scaled by log_2_ fold change (*p* value < 0.05), *n* = 2 independent experiments. (B) GSEA enrichment plot of proteomics data highlighting protein enrichment for radial glial cells in NSC11 sgCARM1 and sgSCR cells. (C) heatmap of differentially expressed proteins (*p* value < 0.05) that belong to the above-mentioned GSEA pathway scaled by log_2_ foldchange of sgCARM1 cells vs. sgSCR. (D) Scatterplot of log_2_ fold change (averaged between sgCARM1-1 and sgCARM1-2) of RNA-seq and proteomics data, only including genes differentially expressed in both sgCARM1-1 and sgCARM1-2 at the RNA and protein levels. (E) Scatterplot of ranked genes/proteins based on their combined average log_2_ fold change between RNA and protein.

**Figure 4.**
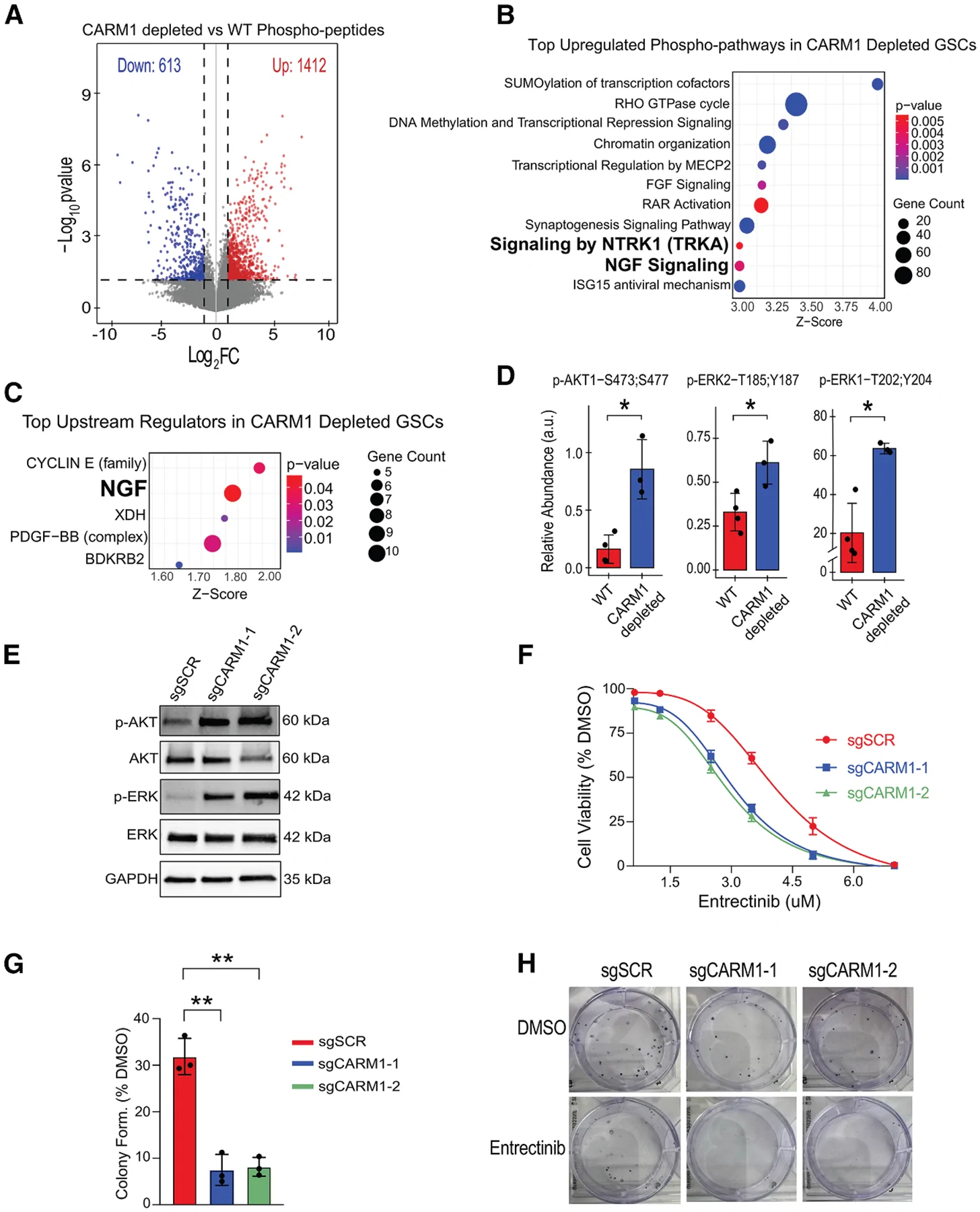
Loss of CARM1 drives NGFR/NTRK signaling in glioma stem-like cells (A) Volcano plot of differentially phosphorylated peptides between NSC11 WT and CARM1-depleted cells (sgCARM1-1) with *n* = 3–4 (*p* value < 0.05). (B and C) Dot plots of Qiagen IPA analysis of top upregulated pathways and (C)top upstream regulators ranked by *Z* score. (D) Bar plots of phosphorylation found on NGFR/NTRK targets AKT1 and ERK1/2 (statistical analysis performed with Welch’s or Student’s *t* test). (E) Western blot of *p*-AKT and *p*-ERK signaling after 6 h of EGF and bFGF starvation. (F) Cell viability of NSC11 GSCs after 96 h of entrectinib measured with Cell Titer Glo 2.0, *n* = 3, dose-response curves were generated using Prism (v. 10) non-linear regression analysis and data show mean ± SEM. (G and H) Colony formation assay of NSC11 GSCs treated for 72 h with entrectinib, *n* = 3, data show mean ± SD. Statistical analysis was performed with Student’s t tests *p < 0.05; **p < 0.01; ***p < 0.001; ****p < 0.0001.

**Figure 5.**
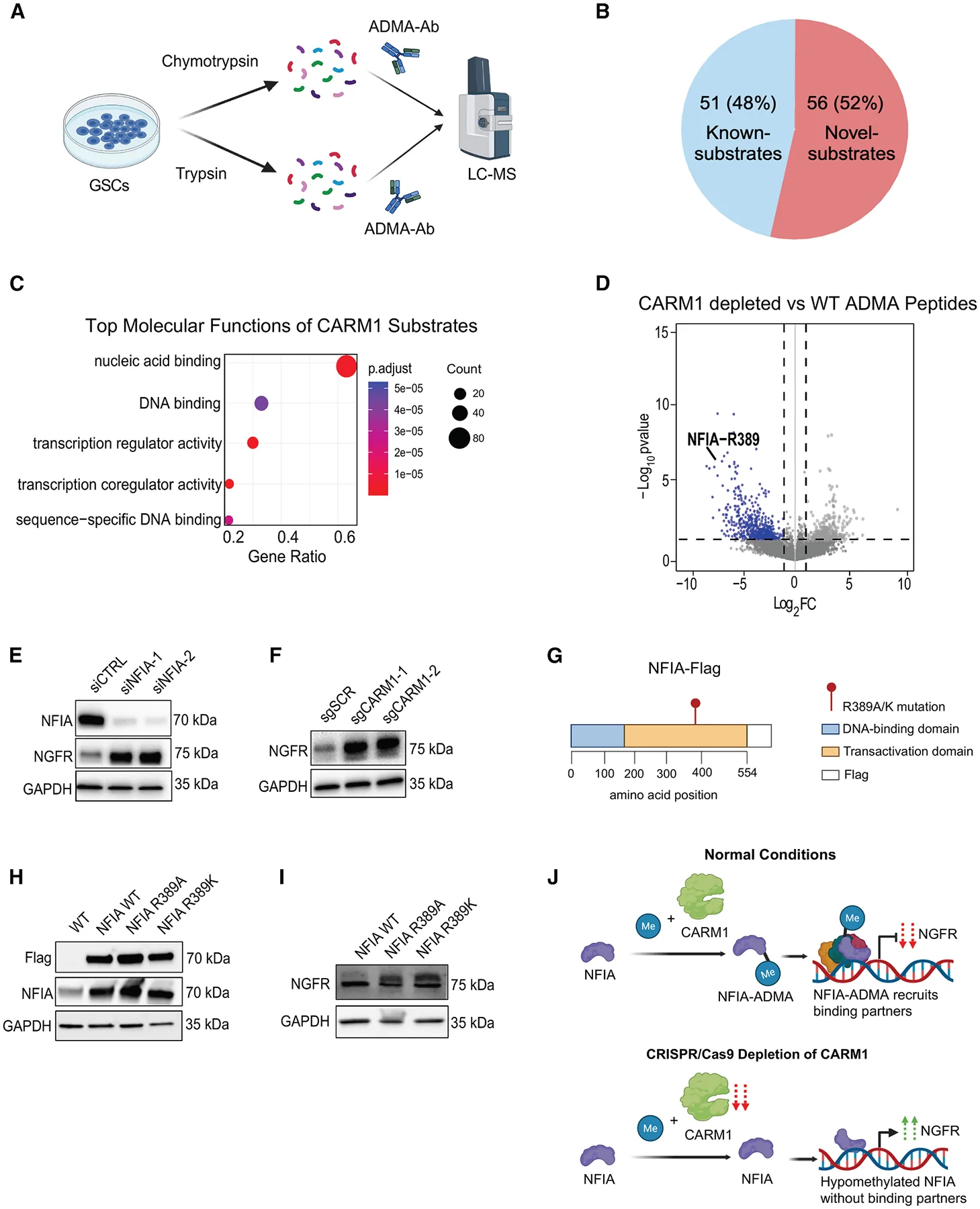
CARM1-dependent methylation of NFIA R389 contributes to repression of NGFR expression in glioma stem-like cells (A) Mass spectrometry schematic of approach to identify CARM1 asymmetric di-methyl arginine (ADMA) substrates in NSC11 GSCs WT vs. CARM1-depleted (sgCARM1-1), *n* = 3–4. (B) Pie chart of identified CARM1 substrates. (C) Dot plot of Gene Ontology molecular functions of statistically significant (*p* value < 0.05) CARM1 substrates and (D) volcano plot of differentially expressed CARM1 ADMA peptides, labeling key substrates (i.e., NFIA). (E and F) Western blots of NFIA and NGFR expression after knockdown of NFIA and (F) of NGFR expression post CARM1 depletion. (G) Schematic of NFIA mutation design, highlighting the R389 location, DNA binding domain, and transactivation domain. NSC11 GSCs were transduced with lentivirus for either FLAG-tagged NFIA R389A, NFIA R389K, or wild-type NFIA. (H) Transduced NFIA expression was validated with western blot of FLAG and NFIA. (I) Western blot of NGFR in NSC11 NFIA WT, NFIA R389A, and NFIA R389K *n* = 2 independent experiments. (J) Schematic of proposed mechanism of NFIA R389 ADMA-dependent repression of NGFR. In the proposed mechanism, CARM1 normally represses NGFR expression by methylating NFIA on R389 which likely functions to help recruit various binding partners (i.e., co-repressors). However, after depletion of CARM1, we propose that NFIA is no longer methylated at R389 and its ability to recruit other transcription regulators is likely disrupted, overall leading to increased NGFR expression.

**Figure 6.**
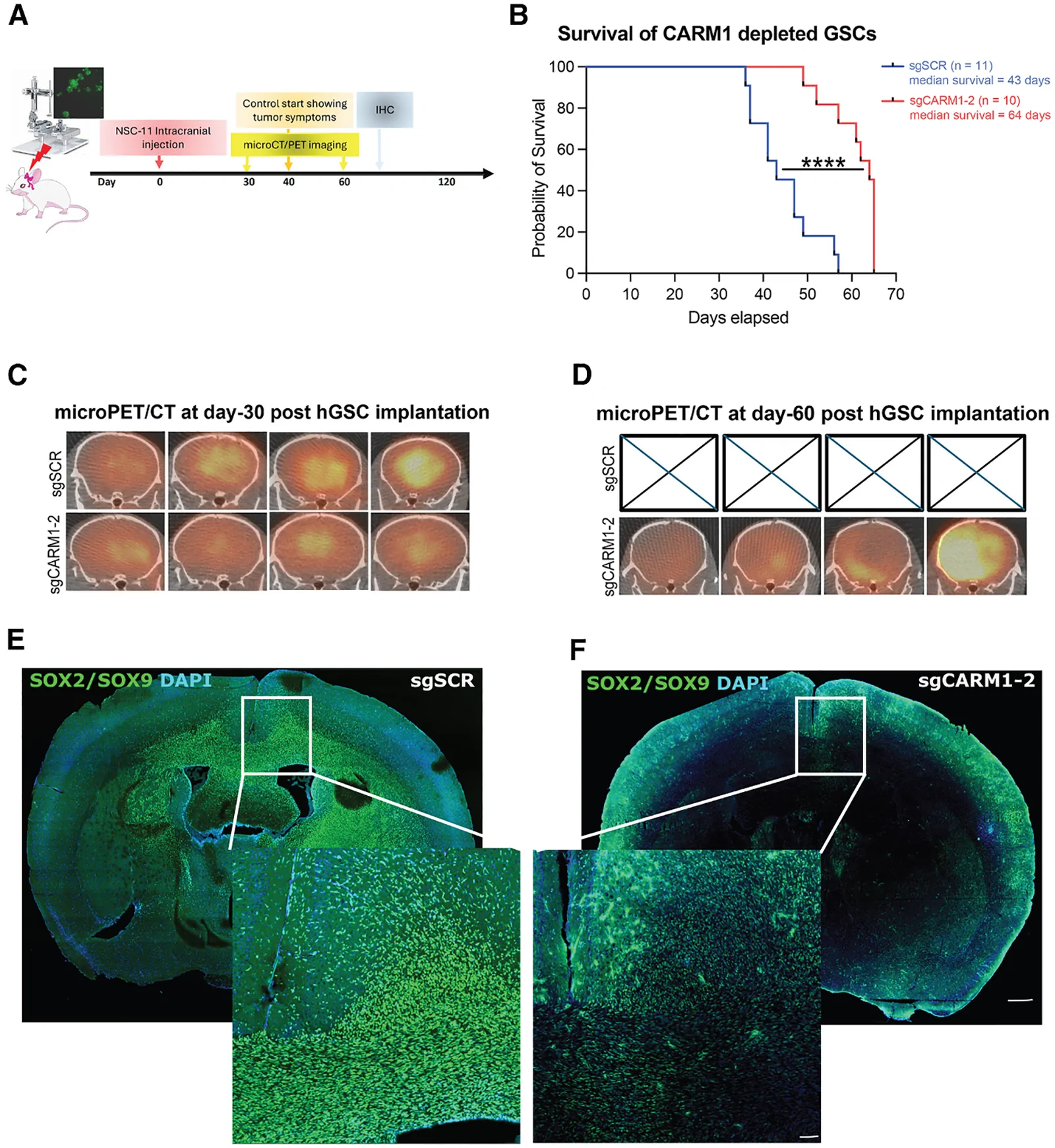
Loss of CARM1 suppresses GSC *in vivo* tumorigenicity (A) Timeline of *in vivo* experiments. (B) Kaplan-Meier survival analysis of NSG mice intracranially implanted with patient-derived GSCs (NSC11) expressing sgSCR or sgCARM1-2 (2 × 10^5^ cells per mouse). A total of 11 mice were included in the sgSCR group and 10 mice in the sgCARM1-2 group. Survival was significantly prolonged in the sgCARM1-2 group (*p* = 0.0001, log rank [Mantel-Cox] test). (C) Representative microPET/CT images showing [^18^F] fluorodeoxyglucose (FDG) uptake in the brains of mice bearing sgSCR and sgCARM1-2 tumors at 30 days post-implantation. (D) microPET/CT imaging at 60 days post-implantation showing that mice in the sgCARM1-2 group (*n* = 4 remaining) were still alive, whereas all mice in the sgSCR group had reached endpoint. (E and F) Representative fluorescence images of intracranial tumors derived from NSC11 sgSCR and sgCARM1-2 cells. sgSCR tumor cells are more abundant and exhibit a more invasive phenotype, with tumor cells spreading along white matter tracts into the contralateral hemisphere. Glioma stem cell markers SOX2 and SOX9 are shown in green, and nuclei are stained with DAPI (blue). Scale bars: 400 μm (overview) and 100 μm (zoomed images).

**Table:** KEY RESOURCES TABLE

REAGENT or RESOURCE	SOURCE	IDENTIFIER
Antibodies		
Anti-CARM1 antibody	Thermo Fisher Scientific	Cat# A300-421A
Anti-NGFR/p75NTR antibody	Cell Signaling Technology	Cat# 8238
Anti-ERK1/2 antibody	Cell Signaling Technology	Cat# 9102
Anti-phospho-ERK1/2 (Thr202/Tyr204) antibody	Cell Signaling Technology	Cat# 4370
Anti-NFIA antibody	Cell Signaling Technology	Cat# 69375
Anti-AKT antibody	Cell Signaling Technology	Cat# 4691
Anti-phospho-AKT (Ser473) antibody	Cell Signaling Technology	Cat# 9271
Anti-GAPDH antibody	Santa Cruz Biotechnology	Cat# sc-47724
Alexa Fluor 488-conjugated anti-SOX2 antibody	eBioscience	N/A
Alexa Fluor 488-conjugated anti-SOX9 antibody	Novus Biologicals	N/A
Anti-FLAG antibody	Cell Signaling Technology	14793S
Chemicals, peptides, and recombinant proteins		
Entrectinib	MedChemExpress	Cat# HY-12678
Capivasertib	MedChemExpress	Cat# HY-15431
Blasticidin S	Thermo Fisher Scientific	Cat# A1113903
Puromycin	Sigma-Aldrich	Cat# P9620
Lipofectamine RNAiMAX transfection reagent	Thermo Fisher Scientific	N/A
Lentivirus concentration reagent	System Biosciences	Cat# LV810A-1
FITC Annexin V	BD Pharmingen	Cat# 556419
Annexin V binding buffer	BD Pharmingen	Cat# 51-66121E
Propidium iodide	EMD Millipore	Cat# 537060
DAPI	Invitrogen	Cat# D3571
RNase A	EMD Millipore	Cat# 70856
Pierce Universal Nuclease	Thermo Fisher Scientific	Cat# 88701
Titanium dioxide beads	GL Sciences	Cat# 5010-21315
Oasis HLB C18 resin	Waters	N/A
ReproSil-Pur C18-AQ, 3 micrometer resin	Dr. Maisch GmbH	N/A
Sequencing-grade trypsin	Promega	N/A
Sequencing-grade chymotrypsin	Promega	N/A
Poly-L-lysine	Sigma-Aldrich	N/A
[18F]fluorodeoxyglucose ([18F]FDG)	N/A	N/A
Critical commercial assays		
CellTiter-Glo 2.0 Cell Viability Assay	Promega	Cat# G9242
PTMScan Asymmetric Di-Methyl Arginine Motif Antibody Bead Conjugate	Cell Signaling Technology	Cat# 18303S
S-Trap protein sample processing filters	Protifi	N/A
RNeasy kit	QIAGEN	N/A
Qubit RNA quantification assay	Thermo Fisher Scientific	N/A
BCA protein assay	Thermo Fisher Scientific	N/A
Deposited data		
Mass spectrometry proteomics data	This paper; ProteomeXchange Consortium via PRIDE	PXD080227
Raw RNA-sequencing data	This paper; NCBI Sequence Read Archive	BioProject: PRJNA1237514
Processed gene-expression data	This paper; NCBI Gene Expression Omnibus	GEO: GSE292789
TCGA glioblastoma and lower-grade glioma datasets	NCI Genomic Data Commons; UCSC Xena	TCGA-GBM; TCGA-LGG; https://xenabrowser.net
Human reference genome, GRCh38 primary assembly	Genome Reference Consortium; NCBI	GRCh38; GenBank: GCA_000001405.15
GENCODE human gene annotation, release 42	GENCODE	Release 42; https://www.gencodegenes.org/human/release_42.html
Experimental models: cell lines		
Human: NSC11 patient-derived glioma stem-like cells	Frederick Lang laboratory, MD Anderson Cancer Center	NSC11
Human: NSC20 patient-derived glioma stem-like cells	Frederick Lang laboratory, MD Anderson Cancer Center	NSC20
Human: HEK293T cells	N/A	N/A
Experimental models: organisms/strains		
Mouse: NOD-SCID gamma (NSG): NOD.Cg-Prkdcscid Il2rgtm1Wjl/SzJ	The Jackson Laboratory	JAX: 005557; RRID: IMSR_JAX:005557
Oligonucleotides		
Non-targeting sgRNA control (sgSCR)	This paper	N/A
sgCARM1-1 target sequence: AGCACGGAAAATCTACGCGG	This paper	N/A
sgCARM1-2 target sequence: TGGAGCACGGAAAATCTACG	This paper	N/A
Non-targeting siRNA control (siCTRL)	Thermo Fisher Scientific	N/A
siNFIA-1: sense CGAAAACGAAAAUACUUCAtt; antisense UGAAGUAUUUUCGUUUUCGgg	Thermo Fisher Scientific	Cat# s9477
siNFIA-2: sense CACAUCACCCAUUAUCCAtt; antisense UGGAUAAUGGGUGAUGUCGgg	Thermo Fisher Scientific	Cat# s9478
siNGFR: sense CCACAGCAGGUGUCAUAUAtt; antisense UAUAUGACACCUCUGUGGtg	Thermo Fisher Scientific	Cat# s9540
Recombinant DNA		
Plasmid: lentiCas9-Blast	Addgene	Addgene plasmid #52962
Plasmid: pXPR_050	Addgene	Addgene plasmid #96925
Plasmid: pXPR_050-sgSCR	This paper	Available from the Lead Contact
Plasmid: pXPR_050-sgCARM1-1	This paper	Available from the Lead Contact
Plasmid: pXPR_050-sgCARM1-2	This paper	Available from the Lead Contact
Lentiviral plasmid: FLAG-NFIA WT	This paper; VectorBuilder	Sequence in Table S5; available from the Lead Contact
Lentiviral plasmid: FLAG-NFIA R389A	This paper; VectorBuilder	Sequence in Table S5; available from the Lead Contact
Lentiviral plasmid: FLAG-NFIA R389K	This paper; VectorBuilder	Sequence in Table S5; available from the Lead Contact
Software and algorithms		
CRISPick	Broad Institute	https://portals.broadinstitute.org/gppx/crispick/public
Geneious Prime	Dotmatics	https://www.geneious.com
VectorBuilder Design Studio	VectorBuilder	https://en.vectorbuilder.com
FlowJo	BD Biosciences	https://www.flowjo.com
GraphPad Prism, version 10	GraphPad Software	https://www.graphpad.com/scientific-software/prism/
R, version 4.3.1	R Foundation for Statistical Computing	https://www.r-project.org
clusterProfiler	Bioconductor	https://bioconductor.org/packages/clusterProfiler
UCSC Xena Browser	UCSC	https://xenabrowser.net
survival R package	CRAN	https://cran.r-project.org/package=survival
survminer R package	CRAN	https://cran.r-project.org/package=survminer
FragPipe, version 22	FragPipe development team	https://fragpipe.nesvilab.org
EpiProfile, version 2.0	EpiProfile development team	https://github.com/zfyuan/EpiProfile2.0
nf-core/rnaseq, version 3.14.0	nf-core	https://nf-co.re/rnaseq/3.14.0
zFPKM	Hart et al.	https://github.com/stevenhartlab/ExpressionValues
DESeq2	Bioconductor	https://bioconductor.org/packages/DESeq2
Ingenuity Pathway Analysis	QIAGEN	https://digitalinsights.qiagen.com/IPA
ggplot2	The ggplot2 development team	https://ggplot2.tidyverse.org
Molecular Signatures Database (MSigDB), C2 collection	Broad Institute	https://www.gsea-msigdb.org/gsea/msigdb
Gene Ontology	Gene Ontology Consortium	https://geneontology.org
Inveon Acquisition Workplace	Siemens	N/A
Inveon Research Workplace	Siemens	N/A
AEON Analytics Software	Coluxa	N/A
Other		
INVEON PET/SPECT/CT Trimodal System	Siemens	N/A
AEON Cytoscanner	Coluxa	N/A
nanoElute 2 liquid chromatography system	Bruker	N/A
timsTOF HT mass spectrometer	Bruker	N/A
Dionex UltiMate 3000 RSLC system	Thermo Fisher Scientific	N/A
Orbitrap Fusion Lumos mass spectrometer	Thermo Fisher Scientific	N/A
NovaSeq X sequencing system	Illumina	N/A
Qubit fluorometer	Thermo Fisher Scientific	N/A
NanoDrop spectrophotometer	Thermo Fisher Scientific	N/A
C18 trap cartridge	Thermo Fisher Scientific	Cat# 174500
PepSep XTREME analytical column, 25 cm × 150 micrometer × 1.5 micrometer	Bruker	Cat# 1893476

## Highlights

- CARM1 depletion shifts glioma stem-like cells toward a radial glial-like state
- Loss of CARM1 increases NGFR/NTRK signaling and pathway dependence
- NFIA is a CARM1 substrate that contributes to repression of NGFR
- CARM1 depletion reduces glioma tumorigenicity in orthotopic xenografts

## References

[1] Poon MTC, Sudlow CLM, Figueroa JD, and Brennan PM. (2020). Longer-term (>/= 2 years) survival in patients with glioblastoma in population-based studies pre- and post-2005: a systematic review and meta-analysis. Sci. Rep. 10, 11622. 10.1038/s41598-020-68011-4. PMC7363854 32669604

[2] Weller M, Cloughesy T, Perry JR, and Wick W. (2013). Standards of care for treatment of recurrent glioblastoma–are we there yet? Neuro Oncol. 15, 4–27. 10.1093/neuonc/nos273. PMC3534423 23136223

[3] Prager BC, Bhargava S, Mahadev V, Hubert CG, and Rich JN. (2020). Glioblastoma Stem Cells: Driving Resilience through Chaos. Trends Cancer 6, 223–235. 10.1016/j.trecan.2020.01.009. PMC8779821 32101725

[4] Singh SK, Hawkins C, Clarke ID, Squire JA, Bayani J, Hide T, Henkelman RM, Cusimano MD, and Dirks PB. (2004). Identification of human brain tumour initiating cells. Nature 432, 396–401. 10.1038/nature03128. 15549107

[5] Valor LM, and Hervás-Corpión I. (2020). The Epigenetics of Glioma Stem Cells: A Brief Overview. Front. Oncol. 10, 602378. 10.3389/fonc.2020.602378. PMC7738619 33344253

[6] Phillips RE, Soshnev AA, and Allis CD. (2020). Epigenomic Reprogramming as a Driver of Malignant Glioma. Cancer Cell 38, 647–660. 10.1016/j.ccell.2020.08.008. PMC8248764 32916125

[7] Liau BB, Sievers C, Donohue LK, Gillespie SM, Flavahan WA, Miller TE, Venteicher AS, Hebert CH, Carey CD, Rodig SJ, (2017). Adaptive Chromatin Remodeling Drives Glioblastoma Stem Cell Plasticity and Drug Tolerance. Cell Stem Cell 20, 233–246.e7. 10.1016/j.stem.2016.11.003. PMC5291795 27989769

[8] Bryant J-P, Heiss J, and Banasavadi-Siddegowda YK. (2021). Arginine Methylation in Brain Tumors: Tumor Biology and Therapeutic Strategies. Cells 10, 124. 10.3390/cells10010124. PMC7827394 33440687

[9] Blanc RS, and Richard S. (2017). Arginine Methylation: The Coming of Age. Mol. Cell 65, 8–24. 10.1016/j.molcel.2016.11.003. 28061334

[10] Guccione E, and Richard S. (2019). The regulation, functions and clinical relevance of arginine methylation. Nat. Rev. Mol. Cell Biol. 20, 642–657. 10.1038/s41580-019-0155-x. 31350521

[11] Banasavadi-Siddegowda YK, Russell L, Frair E, Karkhanis VA, Relation T, Yoo JY, Zhang J, Sif S, Imitola J, Baiocchi R, and Kaur B. (2017). PRMT5–PTEN molecular pathway regulates senescence and self-renewal of primary glioblastoma neurosphere cells. Oncogene 36, 263–274. 10.1038/onc.2016.199. PMC5240810 27292259

[12] Zheng D, Chen D, Lin F, Wang X, Lu L, Luo S, Chen J, and Xu X. (2020). LncRNA NNT-AS1 promote glioma cell proliferation and metastases through miR-494-3p/PRMT1 axis. Cell Cycle 19, 1621–1631. 10.1080/15384101.2020.1762037. PMC7469481 32420808

[13] Sachamitr P, Ho JC, Ciamponi FE, Ba-Alawi W, Coutinho FJ, Guilhamon P, Kushida MM, Cavalli FMG, Lee L, Rastegar N, (2021). PRMT5 inhibition disrupts splicing and stemness in glioblastoma. Nat. Commun. 12, 979. 10.1038/s41467-021-21204-5. PMC7881162 33579912

[14] Santos M, Hwang JW, and Bedford MT. (2023). CARM1 arginine methyltransferase as a therapeutic target for cancer. J. Biol. Chem. 299, 105124. 10.1016/j.jbc.2023.105124. PMC10474102 37536629

[15] Wu Q, Bruce AW, Jedrusik A, Ellis PD, Andrews RM, Langford CF, Glover DM, and Zernicka-Goetz M. (2009). CARM1 is Required in Embryonic Stem Cells to Maintain Pluripotency and Resist Differentiation. Stem Cell. 27, 2637–2645. 10.1002/stem.131. PMC4135545 19544422

[16] Torres-Padilla M-E, Parfitt DE, Kouzarides T, and Zernicka-Goetz M. (2007). Histone arginine methylation regulates pluripotency in the early mouse embryo. Nature 445, 214–218. 10.1038/nature05458. PMC3353120 17215844

[17] Rios ÁFL, Tirapelli D.P.d.C., Cirino M.L.d.A., Rodrigues AR, Ramos ES, and Carlotti CG Jr. (2022). Expression of pluripotency-related genes in human glioblastoma. Neurooncol. Adv. 4, vdab163. 10.1093/noajnl/vdab163. PMC8903226 35274101

[18] Wang F, Zhang J, Ke X, Peng W, Zhao G, Peng S, Xu J, Xu B, and Cui H. (2020). WDR5-Myc axis promotes the progression of glioblastoma and neuroblastoma by transcriptional activating CARM1. Biochem. Biophys. Res. Commun. 523, 699–706. 10.1016/j.bbrc.2019.12.101. 31948749

[19] Wan L, Wen H, Li Y, Lyu J, Xi Y, Hoshii T, Joseph JK, Wang X, Loh YHE, Erb MA, (2017). ENL links histone acetylation to oncogenic gene expression in acute myeloid leukaemia. Nature 543, 265–269. 10.1038/nature21687. PMC5372383 28241141

[20] McAbee JH, Rath BH, Valdez K, Young DL, Wu X, Shankavaram UT, Camphausen K, and Tofilon PJ. (2019). Radiation Drives the Evolution of Orthotopic Xenografts Initiated from Glioblastoma Stem-like Cells. Cancer Res. 79, 6032–6043. 10.1158/0008-5472.CAN-19-2452. PMC6891212 31615806

[21] Ishino Y, Shimizu S, Tohyama M, and Miyata S. (2022). Coactivator-associated arginine methyltransferase 1 controls oligodendrocyte differentiation in the corpus callosum during early brain development. Dev. Neurobiol. 82, 245–260. 10.1002/dneu.22871. 35220691

[22] Lim CS, and Alkon DL. (2017). Inhibition of coactivator-associated arginine methyltransferase 1 modulates dendritic arborization and spine maturation of cultured hippocampal neurons. J. Biol. Chem. 292, 6402–6413. 10.1074/jbc.m117.775619. PMC5391767 28264928

[23] Bauer UM, Daujat S, Nielsen SJ, Nightingale K, and Kouzarides T. (2002). Methylation at arginine 17 of histone H3 is linked to gene activation. EMBO Rep. 3, 39–44. 10.1093/embo-reports/kvf013. PMC1083932 11751582

[24] Wu J, Cui N, Wang R, Li J, and Wong J. (2012). A Role for CARM1-Mediated Histone H3 Arginine Methylation in Protecting Histone Acetylation by Releasing Corepressors from Chromatin. PLoS One 7, e34692. 10.1371/journal.pone.0034692. PMC3377634 22723830

[25] Park S, Cho JH, Kim JH, and Kim JA. (2024). Histone lysine methylation modifiers controlled by protein stability. Exp. Mol. Med. 56, 2127–2144. 10.1038/s12276-024-01329-5. PMC11541785 39394462

[26] Chung N, Bogliotti YS, Ding W, Vilarino M, Takahashi K, Chitwood JL, Schultz RM, and Ross PJ. (2017). Active H3K27me3 demethylation by KDM6B is required for normal development of bovine preimplantation embryos. Epigenetics 12, 1048–1056. 10.1080/15592294.2017.1403693. PMC5810760 29160132

[27] Hyun K, Jeon J, Park K, and Kim J. (2017). Writing, erasing and reading histone lysine methylations. Exp. Mol. Med. 49, e324. 10.1038/emm.2017.11. PMC6130214 28450737

[28] Kuo Y-M, and Andrews AJ. (2013). Quantitating the Specificity and Selectivity of Gcn5-Mediated Acetylation of Histone H3. PLoS One 8, e54896. 10.1371/journal.pone.0054896. PMC3578832 23437046

[29] Tsukada Y-I, Fang J, Erdjument-Bromage H, Warren ME, Borchers CH, Tempst P, and Zhang Y. (2006). Histone demethylation by a family of JmjC domain-containing proteins. Nature 439, 811–816. 10.1038/nature04433. 16362057

[30] Buontempo S, Laise P, Hughes JM, Trattaro S, Das V, Rencurel C, and Testa G. (2022). EZH2-Mediated H3K27me3 Targets Transcriptional Circuits of Neuronal Differentiation. Front. Neurosci. 16, 814144. 10.3389/fnins.2022.814144. PMC9133892 35645710

[31] Vezzoli M, de Llobet Cucalon LI, Di Vona C, Morselli M, Montanini B, de la Luna S, Teichmann M, Dieci G, and Ferrari R. (2023). TFIIIC as a Potential Epigenetic Modulator of Histone Acetylation in Human Stem Cells. Int. J. Mol. Sci. 24, 3624. 10.3390/ijms24043624. PMC9961906 36835038

[32] Funk OH, Qalieh Y, Doyle DZ, Lam MM, and Kwan KY. (2022). Postmitotic accumulation of histone variant H3.3 in new cortical neurons establishes neuronal chromatin, transcriptome, and identity. Proc. Natl. Acad. Sci. USA 119, e2116956119. 10.1073/pnas.2116956119. PMC9371731 35930666

[33] Rajagopalan KN, Chen X, Weinberg DN, Chen H, Majewski J, Allis CD, and Lu C. (2021). Depletion of H3K36me2 recapitulates epigenomic and phenotypic changes induced by the H3.3K36M oncohistone mutation. Proc. Natl. Acad. Sci. USA 118, e2021795118. 10.1073/pnas.2021795118. PMC7936350 33619101

[34] Zaghi M, Broccoli V, and Sessa A. (2019). H3K36 Methylation in Neural Development and Associated Diseases. Front. Genet. 10, 1291. 10.3389/fgene.2019.01291. PMC6962298 31998360

[35] Joseph-Chowdhury JN, Stransky S, Graff S, Cutler R, Young D, Kim JS, Madrid-Aliste C, Aguilan JT, Nieves E, Sun Y, (2022). Global Level Quantification of Histone Post-Translational Modifications in a 3D Cell Culture Model of Hepatic Tissue. J. Vis. Exp. 10.3791/63606 PMC10042382 35604167

[36] Cattaneo P, Kunderfranco P, Greco C, Guffanti A, Stirparo GG, Rusconi F, Rizzi R, Di Pasquale E, Locatelli SL, Latronico MVG, (2016). DOT1L-mediated H3K79me2 modification critically regulates gene expression during cardiomyocyte differentiation. Cell Death Differ. 23, 555–564. 10.1038/cdd.2014.199. PMC4986629 25526092

[37] Ferrari F, Arrigoni L, Franz H, Izzo A, Butenko L, Trompouki E, Vogel T, and Manke T. (2020). DOT1L-mediated murine neuronal differentiation associates with H3K79me2 accumulation and preserves SOX2-enhancer accessibility. Nat. Commun. 11, 5200. 10.1038/s41467-020-19001-7. PMC7562744 33060580

[38] Florio M, Albert M, Taverna E, Namba T, Brandl H, Lewitus E, Haffner C, Sykes A, Wong FK, Peters J, (2015). Human-specific gene ARHGAP11B promotes basal progenitor amplification and neocortex expansion. Science 347, 1465–1470. 10.1126/science.aaa1975. 25721503

[39] Miranda-Negrón Y, and García-Arrarás JE. (2022). Radial glia and radial glia-like cells: Their role in neurogenesis and regeneration. Front. Neurosci. 16, 1006037. 10.3389/fnins.2022.1006037. PMC9708897 36466166

[40] Wang R, Sharma R, Shen X, Laughney AM, Funato K, Clark PJ, Shpokayte M, Morgenstern P, Navare M, Xu Y, (2020). Adult human glioblastomas harbor radial glia-like cells. Stem Cell Rep. 14, 338–350. 10.1016/j.stemcr.2020.01.007. PMC7014025 32004492

[41] Bhaduri A, Di Lullo E, Jung D, Müller S, Crouch EE, Espinosa CS, Ozawa T, Alvarado B, Spatazza J, Cadwell CR, (2020). Outer Radial Glia-like Cancer Stem Cells Contribute to Heterogeneity of Glioblastoma. Cell Stem Cell 26, 48–63.e6. 10.1016/j.stem.2019.11.015. PMC7029801 31901251

[42] Lu X, Maturi NP, Jarvius M, Yildirim I, Dang Y, Zhao L, Xie Y, Tan EJ, Xing P, Larsson R, (2022). Cell-lineage controlled epigenetic regulation in glioblastoma stem cells determines functionally distinct subgroups and predicts patient survival. Nat. Commun. 13, 2236. 10.1038/s41467-022-29912-2. PMC9038925 35469026

[43] Alcantara Llaguno SR, Wang Z, Sun D, Chen J, Xu J, Kim E, Hatanpaa K, Raisanen J, Burns D, Johnson J, and Parada L. (2015). Adult Lineage-Restricted CNS Progenitors Specify Distinct Glioblastoma Subtypes. Cancer Cell 28, 429–440. 10.1016/j.ccell.2015.09.007. PMC4607935 26461091

[44] Bentley CA, and Lee K-F. (2000). p75 Is Important for Axon Growth and Schwann Cell Migration during Development. J. Neurosci. 20, 7706–7715. 10.1523/jneurosci.20-20-07706.2000. PMC6772891 11027232

[45] Boiko AD, Razorenova OV, van de Rijn M, Swetter SM, Johnson DL, Ly DP, Butler PD, Yang GP, Joshua B, Kaplan MJ, (2010). Human melanoma-initiating cells express neural crest nerve growth factor receptor CD271. Nature 466, 133–137. 10.1038/nature09161. PMC2898751 20596026

[46] Barker PA. (2004). p75NTR is positively promiscuous: novel partners and new insights. Neuron 42, 529–533. 10.1016/j.neuron.2004.04.001. 15157416

[47] Harada C, Guo X, Namekata K, Kimura A, Nakamura K, Tanaka K, Parada LF, and Harada T. (2011). Glia- and neuron-specific functions of TrkB signalling during retinal degeneration and regeneration. Nat. Commun. 2, 189. 10.1038/ncomms1190. PMC3105320 21304518

[48] Cocco E, Scaltriti M, and Drilon A. (2018). NTRK fusion-positive cancers and TRK inhibitor therapy. Nat. Rev. Clin. Oncol. 15, 731–747. 10.1038/s41571-018-0113-0. PMC6419506 30333516

[49] Grogan PT, Deming DA, Helgager J, Ruszkiewicz T, Baskaya MK, Howard SP, and Robins HI. (2022). Entrectinib demonstrates prolonged efficacy in an adult case of radiation-refractory NTRK fusion glioblastoma. Neurooncol. Adv. 4, vdac046. 10.1093/noajnl/vdac046. PMC9167633 35673607

[50] Fischer H, Ullah M, de la Cruz CC, Hunsaker T, Senn C, Wirz T, Wagner B, Draganov D, Vazvaei F, Donzelli M, (2020). Entrectinib, a TRK/ROS1 inhibitor with anti-CNS tumor activity: differentiation from other inhibitors in its class due to weak interaction with P-glycoprotein. Neuro Oncol. 22, 819–829. 10.1093/neuonc/noaa052. PMC7283026 32383735

[51] Desai AV, Robinson GW, Gauvain K, Basu EM, Macy ME, Maese L, Whipple NS, Sabnis AJ, Foster JH, Shusterman S, (2022). Entrectinib in children and young adults with solid or primary CNS tumors harboring NTRK, ROS1, or ALK aberrations (STARTRK-NG). Neuro Oncol. 24, 1776–1789. 10.1093/neuonc/noac087. PMC9527518 35395680

[52] Veazey KJ, Cheng D, Lin K, Villarreal OD, Gao G, Perez-Oquendo M, Van HT, Stratton SA, Green M, Xu H, (2020). CARM1 inhibition reduces histone acetyltransferase activity causing synthetic lethality in CREBBP/EP300-mutated lymphomas. Leukemia 34, 3269–3285. 10.1038/s41375-020-0908-8. PMC7688486 32576962

[53] Pham HQH, Tao X, and Yang Y. (2023). Protein arginine methylation in transcription and epigenetic regulation. Front. Epigenet. Epigenom. 1, 1245832. 10.3389/freae.2023.1245832.

[54] Yang Y, McBride K, Hensley S, Lu Y, Chedin F, and Bedford M. (2014). Arginine methylation facilitates the recruitment of TOP3B to chromatin to prevent R loop accumulation. Mol. Cell 53, 484–497. 10.1016/j.molcel.2014.01.011. PMC3959860 24507716

[55] Nie M, Wang Y, Guo C, Li X, Wang Y, Deng Y, Yao B, Gui T, Ma C, Liu M, (2018). CARM1-mediated methylation of protein arginine methyltransferase 5 represses human γ-globin gene expression in erythroleukemia cells. J. Biol. Chem. 293, 17454–17463. 10.1074/jbc.RA118.004028. PMC6231142 30257864

[56] Lorton BM, Harijan RK, Burgos ES, Bonanno JB, Almo SC, and Shechter D. (2020). A Binary Arginine Methylation Switch on Histone H3 Arginine 2 Regulates Its Interaction with WDR5. Biochemistry 59, 3696–3708. 10.1021/acs.biochem.0c00035. PMC7529705 32207970

[57] Shishkova E, Zeng H, Liu F, Kwiecien NW, Hebert AS, Coon JJ, and Xu W. (2017). Global mapping of CARM1 substrates defines enzyme specificity and substrate recognition. Nat. Commun. 8, 15571. 10.1038/ncomms15571. PMC5458078 28537268

[58] Maron MI, Lehman SM, Gayatri S, DeAngelo JD, Hegde S, Lorton BM, Sun Y, Bai DL, Sidoli S, Gupta V, (2021). Independent transcriptomic and proteomic regulation by type I and II protein arginine methyltransferases. iScience 24, 102971. 10.1016/j.isci.2021.102971. PMC8417332 34505004

[59] Itonaga H, Mookhtiar AK, Greenblatt SM, Liu F, Martinez C, Bilbao D, Rains M, Hamard PJ, Sun J, Umeano AC, (2024). Tyrosine phosphorylation of CARM1 promotes its enzymatic activity and alters its target specificity. Nat. Commun. 15, 3415. 10.1038/s41467-024-47689-4. PMC11035800 38649367

[60] Heng YHE, McLeay RC, Harvey TJ, Smith AG, Barry G, Cato K, Plachez C, Little E, Mason S, Dixon C, (2014). NFIX Regulates Neural Progenitor Cell Differentiation During Hippocampal Morphogenesis. Cereb. Cortex 24, 261–279. 10.1093/cercor/bhs307. PMC3862270 23042739

[61] Piper M, Barry G, Hawkins J, Mason S, Lindwall C, Little E, Sarkar A, Smith AG, Moldrich RX, Boyle GM, (2010). NFIA Controls Telencephalic Progenitor Cell Differentiation through Repression of the Notch Effector Hes1. J. Neurosci. 30, 9127–9139. 10.1523/JNEUROSCI.6167-09.2010. PMC6632468 20610746

[62] Gao G, Hausmann S, Flores NM, Benitez AM, Shen J, Yang X, Person MD, Gayatri S, Cheng D, Lu Y, (2023). The NFIB/CARM1 partnership is a driver in preclinical models of small cell lung cancer. Nat. Commun. 14, 363. 10.1038/s41467-023-35864-y. PMC9870865 36690626

[63] Fraser J, Essebier A, Brown AS, Davila RA, Harkins D, Zalucki O, Shapiro LP, Penzes P, Wainwright BJ, Scott MP, (2020). Common Regulatory Targets of NFIA, NFIX and NFIB during Postnatal Cerebellar Development. Cerebellum 19, 89–101. 10.1007/s12311-019-01089-3. PMC7815246 31838646

[64] Chen KS, Lynton Z, Lim JWC, Robertson T, Gronostajski RM, Bunt J, and Richards LJ. (2021). NFIA and NFIB function as tumour suppressors in high-grade glioma in mice. Carcinogenesis 42, 357–368. 10.1093/carcin/bgaa139. 33346791

[65] Glasgow SM, Carlson JC, Zhu W, Chaboub LS, Kang P, Lee HK, Clovis YM, Lozzi BE, McEvilly RJ, Rosenfeld MG, (2017). Glia-specific enhancers and chromatin structure regulate NFIA expression and glioma tumorigenesis. Nat. Neurosci. 20, 1520–1528. 10.1038/nn.4638. PMC5919190 28892058

[66] Lee J, Hoxha E, and Song HR. (2017). A novel NFIA-NFκB feed-forward loop contributes to glioblastoma cell survival. Neuro Oncol. 19, 524–534. 10.1093/neuonc/now233. PMC5473440 27994064

[67] Glasgow SM, Zhu W, Stolt CC, Huang TW, Chen F, LoTurco JJ, Neul JL, Wegner M, Mohila C, and Deneen B. (2014). Mutual antagonism between Sox10 and NFIA regulates diversification of glial lineages and glioma subtypes. Nat. Neurosci. 17, 1322–1329. 10.1038/nn.3790. PMC4313923 25151262

[68] DeAngelo JD, Maron MI, Roth JS, Hegde S, Silverstein AM, Gupta V, Stransky S, Basken J, Azofeifa J, Query CC, (2025). Productive mRNA chromatin escape is promoted by PRMT5 activity. Mol. Cell 85, 4016–4031.e9. 10.1016/j.molcel.2025.09.021. PMC12744693 41086806

[69] Wei HH, Fan XJ, Hu Y, Tian XX, Guo M, Mao MW, Fang ZY, Wu P, Gao SX, Peng C, (2021). A systematic survey of PRMT interactomes reveals the key roles of arginine methylation in the global control of RNA splicing and translation. Sci. Bull. 66, 1342–1357. 10.1016/j.scib.2021.01.004. 36654156

[70] Jain K, Warmack RA, Debler EW, Hadjikyriacou A, Stavropoulos P, and Clarke SG. (2016). Protein Arginine Methyltransferase Product Specificity Is Mediated by Distinct Active-site Architectures. J. Biol. Chem. 291, 18299–18308. 10.1074/jbc.m116.740399. PMC5000077 27387499

[71] Li WJ, He Y.h., Yang J.j., Hu G.s., Lin Y.a., Ran T, Peng B.l., Xie B.l., Huang M.f., Gao X, (2021). Profiling PRMT methylome reveals roles of hnRNPA1 arginine methylation in RNA splicing and cell growth. Nat. Commun. 12, 1946. 10.1038/s41467-021-21963-1. PMC8007824 33782401

[72] Ben-Porath I, Thomson MW, Carey VJ, Ge R, Bell GW, Regev A, and Weinberg RA. (2008). An embryonic stem cell-like gene expression signature in poorly differentiated aggressive human tumors. Nat. Genet. 40, 499–507. 10.1038/ng.127. PMC2912221 18443585

[73] Suvà ML, Rheinbay E, Gillespie S, Patel A, Wakimoto H, Rabkin S, Riggi N, Chi A, Cahill D, Nahed B, (2014). Reconstructing and reprogramming the tumor-propagating potential of glioblastoma stem-like cells. Cell 157, 580–594. 10.1016/j.cell.2014.02.030. PMC4004670 24726434

[74] Liu Z, Yu K, Chen K, Zhang Y, Dai K, Zhao L, and Zhao P. (2025). USP18 deubiquitinates and stabilizes SOX9 to promote the stemness and malignant progression of glioblastoma. Cell Death Discov. 11, 237. 10.1038/s41420-025-02522-9. PMC12081856 40374599

[75] Swartling FJ, Savov V, Persson A, Chen J, Hackett C, Northcott P, Grimmer M, Lau J, Chesler L, Perry A, (2012). Distinct neural stem cell populations give rise to disparate brain tumors in response to N-MYC. Cancer Cell 21, 601–613. 10.1016/j.ccr.2012.04.012. PMC3360885 22624711

[76] Cheng D, Côté J, Shaaban S, and Bedford MT. (2007). The Arginine Methyltransferase CARM1 Regulates the Coupling of Transcription and mRNA Processing. Mol. Cell 25, 71–83. 10.1016/j.molcel.2006.11.019. 17218272

[77] Yu Z, Chen T, Hébert J, Li E, and Richard S. (2009). A Mouse PRMT1 Null Allele Defines an Essential Role for Arginine Methylation in Genome Maintenance and Cell Proliferation. Mol. Cell Biol. 29, 2982–2996. 10.1128/MCB.00042-09. PMC2681996 19289494

[78] Tee WW, Pardo M, Theunissen TW, Yu L, Choudhary JS, Hajkova P, and Surani MA. (2010). Prmt5 is essential for early mouse development and acts in the cytoplasm to maintain ES cell pluripotency. Genes Dev. 24, 2772–2777. 10.1101/gad.606110. PMC3003195 21159818

[79] Wongtawan T, Taylor JE, Lawson KA, Wilmut I, and Pennings S. (2011). Histone H4K20me3 and HP1α are late heterochromatin markers in development, but present in undifferentiated embryonic stem cells. J. Cell Sci. 124, 1878–1890. 10.1242/jcs.080721. 21576353

[80] Sidoli S, Lu C, Coradin M, Wang X, Karch KR, Ruminowicz C, and Garcia BA. (2017). Metabolic labeling in middle-down proteomics allows for investigation of the dynamics of the histone code. Epigenetics Chromatin 10, 34. 10.1186/s13072-017-0139-z. PMC5501349 28683815

[81] Sidoli S, Lopes M, Lund PJ, Goldman N, Fasolino M, Coradin M, Kulej K, Bhanu NV, Vahedi G, and Garcia BA. (2019). A mass spectrometry-based assay using metabolic labeling to rapidly monitor chromatin accessibility of modified histone proteins. Sci. Rep. 9, 13613. 10.1038/s41598-019-49894-4. PMC6754405 31541121

[82] Wang Z, Zang C, Rosenfeld JA, Schones DE, Barski A, Cuddapah S, Cui K, Roh TY, Peng W, Zhang MQ, and Zhao K. (2008). Combinatorial patterns of histone acetylations and methylations in the human genome. Nat. Genet. 40, 897–903. 10.1038/ng.154. PMC2769248 18552846

[83] Luo M, Bai J, Liu B, Yan P, Zuo F, Sun H, Sun Y, Xu X, Song Z, Yang Y, (2019). H3K18ac Primes Mesendodermal Differentiation upon Nodal Signaling. Stem Cell Rep. 13, 642–656. 10.1016/j.stemcr.2019.08.016. PMC6830056 31564646

[84] Mostaqul Huq MD, Gupta P, Tsai N, White R, Parker MG, and Wei L. (2006). Suppression of receptor interacting protein 140 repressive activity by protein arginine methylation. EMBO J. 25, 5094–5104. 10.1038/sj.emboj.7601389. PMC1630415 17053781

[85] Iwasaki H, Kovacic JC, Olive M, Beers JK, Yoshimoto T, Crook MF, Tonelli LH, and Nabel EG. (2010). Disruption of Protein Arginine N -Methyltransferase 2 Regulates Leptin Signaling and Produces Leanness In Vivo Through Loss of STAT3 Methylation. Circ. Res. 107, 992–1001. 10.1161/circresaha.110.225326. PMC2997704 20798359

[86] Shen L, Ma X, Wang Y, Wang Z, Zhang Y, Pham HQH, Tao X, Cui Y, Wei J, Lin D, (2024). Loss-of-function mutation in PRMT9 causes abnormal synapse development by dysregulation of RNA alternative splicing. Nat. Commun. 15, 2809. 10.1038/s41467-024-47107-9. PMC10984984 38561334

[87] Perez-Riverol Y, Bai J, Bandla C, García-Seisdedos D, Hewapathirana S, Kamatchinathan S, Kundu DJ, Prakash A, Frericks-Zipper A, Eisenacher M, (2022). The PRIDE database resources in 2022: a hub for mass spectrometry-based proteomics evidences. Nucleic Acids Res. 50, D543–D552. 10.1093/nar/gkab1038. PMC8728295 34723319

[88] McCord AM, Jamal M, Williams ES, Camphausen K, and Tofilon PJ. (2009). CD133+ glioblastoma stem-like cells are radiosensitive with a defective DNA damage response compared with established cell lines. Clin. Cancer Res. 15, 5145–5153. 10.1158/1078-0432.ccr-09-0263. PMC6290462 19671863

[89] Behnan J, Stangeland B, Hosainey SAM, Joel M, Olsen TK, Micci F, Glover JC, Isakson P, and Brinchmann JE. (2017). Differential propagation of stroma and cancer stem cells dictates tumorigenesis and multipotency. Oncogene 36, 570–584. 10.1038/onc.2016.230. PMC5290038 27345406

[90] Dubey A, Yamashita E, Stangeland B, Abbas I, Fooksman D, Harris RA, Palmer GM, Koba WR, Zhang J, Himes BT, (2025). Brain tumors induce widespread disruption of calvarial bone and alteration of skull marrow immune landscape. Nat. Neurosci. 28, 2231–2246. 10.1038/s41593-025-02064-4. PMC12586139 41044343

[91] Ran FA, Hsu PD, Wright J, Agarwala V, Scott DA, and Zhang F. (2013). Genome engineering using the CRISPR-Cas9 system. Nat. Protoc. 8, 2281–2308. 10.1038/nprot.2013.143. PMC3969860 24157548

[92] Franken NAP, Rodermond HM, Stap J, Haveman J, and van Bree C. (2006). Clonogenic assay of cells in vitro. Nat. Protoc. 1, 2315–2319. 10.1038/nprot.2006.339. 17406473

[93] Mayoral J, Tomita T, Tu V, Aguilan JT, Sidoli S, and Weiss LM. (2020). Toxoplasma gondii PPM3C, a secreted protein phosphatase, affects parasitophorous vacuole effector export. PLoS Pathog. 16, e1008771. 10.1371/journal.ppat.1008771. PMC7793252 33370417

[94] Stransky S, Cutler R, Aguilan J, Nieves E, and Sidoli S. (2022). Investigation of reversible histone acetylation and dynamics in gene expression regulation using 3D liver spheroid model. Epigenetics Chromatin 15, 35. 10.1186/s13072-022-00470-7. PMC9677638 36411440

[95] Yu G, Wang LG, Han Y, and He QY. (2012). clusterProfiler: an R Package for Comparing Biological Themes Among Gene Clusters. OMICS A J. Integr. Biol. 16, 284–287. 10.1089/omi.2011.0118. PMC3339379 22455463

[96] Goldman MJ, Craft B, Hastie M, Repečka K, McDade F, Kamath A, Banerjee A, Luo Y, Rogers D, Brooks AN, (2020). Visualizing and interpreting cancer genomics data via the Xena platform. Nat. Biotechnol. 38, 675–678. 10.1038/s41587-020-0546-8. PMC7386072 32444850

[97] Demichev V, Szyrwiel L, Yu F, Teo GC, Rosenberger G, Niewienda A, Ludwig D, Decker J, Kaspar-Schoenefeld S, Lilley KS, (2022). dia-PASEF data analysis using FragPipe and DIA-NN for deep proteomics of low sample amounts. Nat. Commun. 13, 3944. 10.1038/s41467-022-31492-0. PMC9270362 35803928

[98] Yuan ZF, Sidoli S, Marchione DM, Simithy J, Janssen KA, Szurgot MR, and Garcia BA. (2018). EpiProfile 2.0: A Computational Platform for Processing Epi-Proteomics Mass Spectrometry Data. J. Proteome Res. 17, 2533–2541. 10.1021/acs.jproteome.8b00133. PMC6387837 29790754

[99] Ewels PA, Peltzer A, Fillinger S, Patel H, Alneberg J, Wilm A, Garcia MU, Di Tommaso P, and Nahnsen S. (2020). The nf-core framework for community-curated bioinformatics pipelines. Nat. Biotechnol. 38, 276–278. 10.1038/s41587-020-0439-x. 32055031

[100] Hart T, Komori HK, LaMere S, Podshivalova K, and Salomon DR. (2013). Finding the active genes in deep RNA-seq gene expression studies. BMC Genom. 14, 778. 10.1186/1471-2164-14-778. PMC3870982 24215113

[101] Krämer A, Green J, Pollard J Jr., and Tugendreich S. (2014). Causal analysis approaches in Ingenuity Pathway Analysis. Bioinformatics 30, 523–530. 10.1093/bioinformatics/btt703. PMC3928520 24336805

