# Supplementary material for: Depletion of CARM1 slows in vivo tumorigenicity and rewires developmental programming in glioma stem-like cells
Source: Cell Rep. Author manuscript; Available in PMC 2026 Sep 30. (PMC13625064; doi:10.1016/j.celrep.2026.117785)
Supplement: Supplementary file 1 [file NIHMS2206904-supplement-1.pdf]

Cell Reports, Volume *45*

Supplemental information

Depletion of CARM1 slows *in vivo* tumorigenicity and rewires developmental programming in glioma stem-like cells

Dejauwne L. Young, Stephanie Stransky, Maria G. Molero, Erika Yamashita, Shivangi Pande, Vivek Kumar, Saurabh Singh, Jennifer Aguilan, Ronald Cutler, Wade Koba, Joseph D. DeAngelo, Jacob S. Roth, Jinghang Zhang, Tianfang He, Beata Malachowska, Justin Vercellino, Brett I. Bell, David Shechter, Philip J. Tofilon, Richard E. Phillips, Jinan Behnan, Chandan Guha, and Simone Sidoli

Supplemental Fig. 1

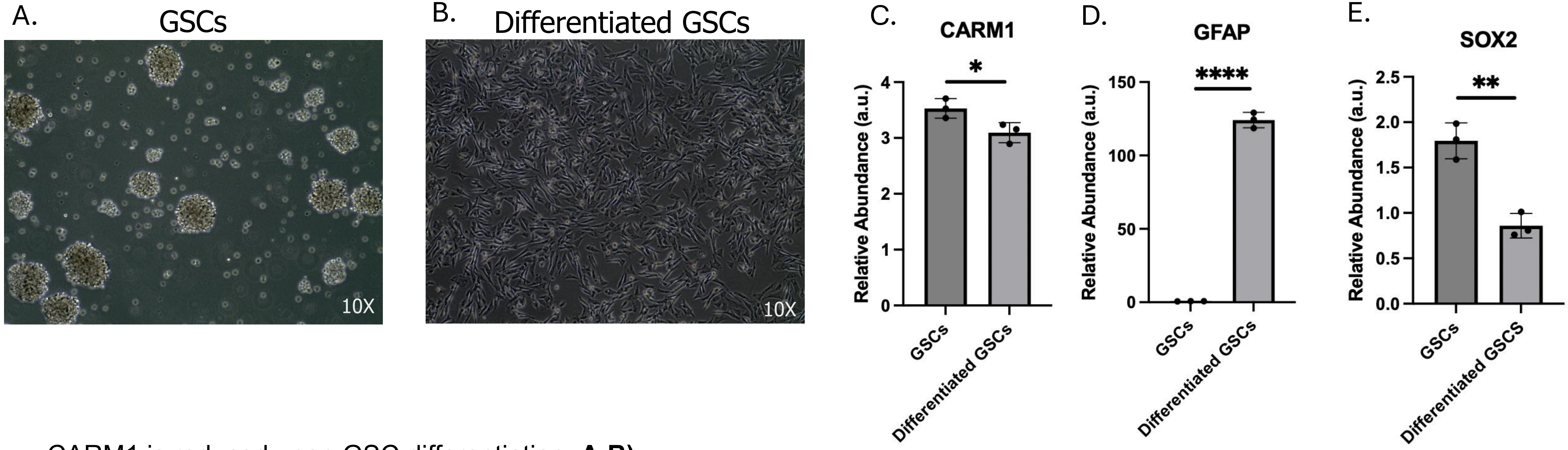

CARM1 is reduced upon GSC differentiation. **A-B)** Images of GSCs under normal conditions and after 7 days of differentiation and **C-D)** mass spectrometry-based protein expression of CARM1, GFAP, and SOX2. Statistical analysis was performed with Student’s or Welch’s T-test *$p < 0.05$; **$p < 0.01$; ***$p < 0.001$; ****$p < 0.0001$

Supplemental Fig. 2

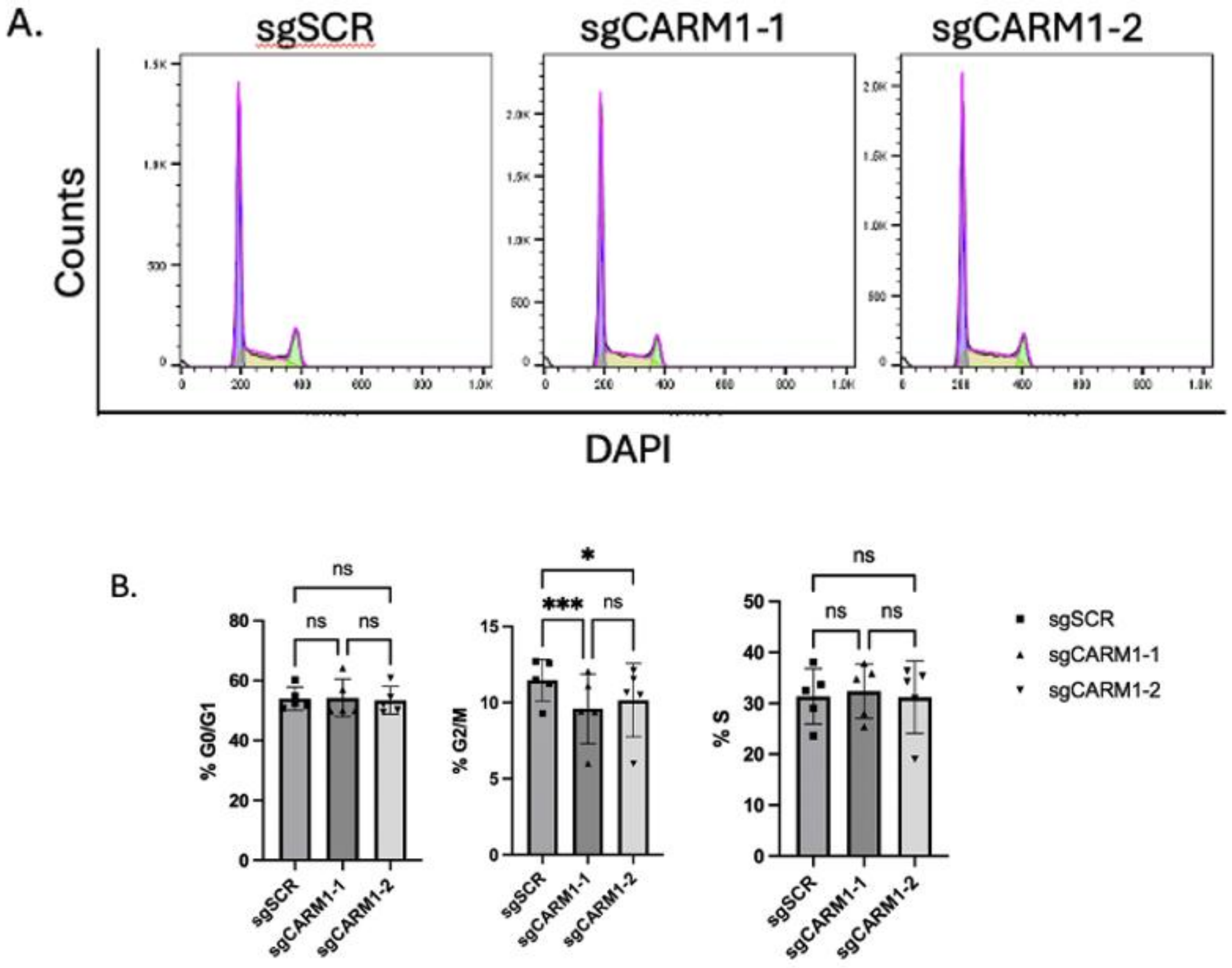

CARM1 loss reduces G2/M population in GSCs. A) Representative DNA content histograms of DAPI-stained nuclei analyzed by flow cytometry in WT, Scramble Control, CARM1_KO_1 and CARM1_KO_2 GSCs. **B)** Quantification of percentage of cells in G0/G1 phase (left), S phase (middle), and G2/M phase (right). Each symbol represents an independent experiment done in triplicate. Data represents mean +/- SD from independent experiments. Statistical significance was determined using two-way ANOVA with Tukey's test. ns, not significant; *$p < 0.05$; **$p < 0.01$; ***$p < 0.001$

Supplementary Fig. 3

A.

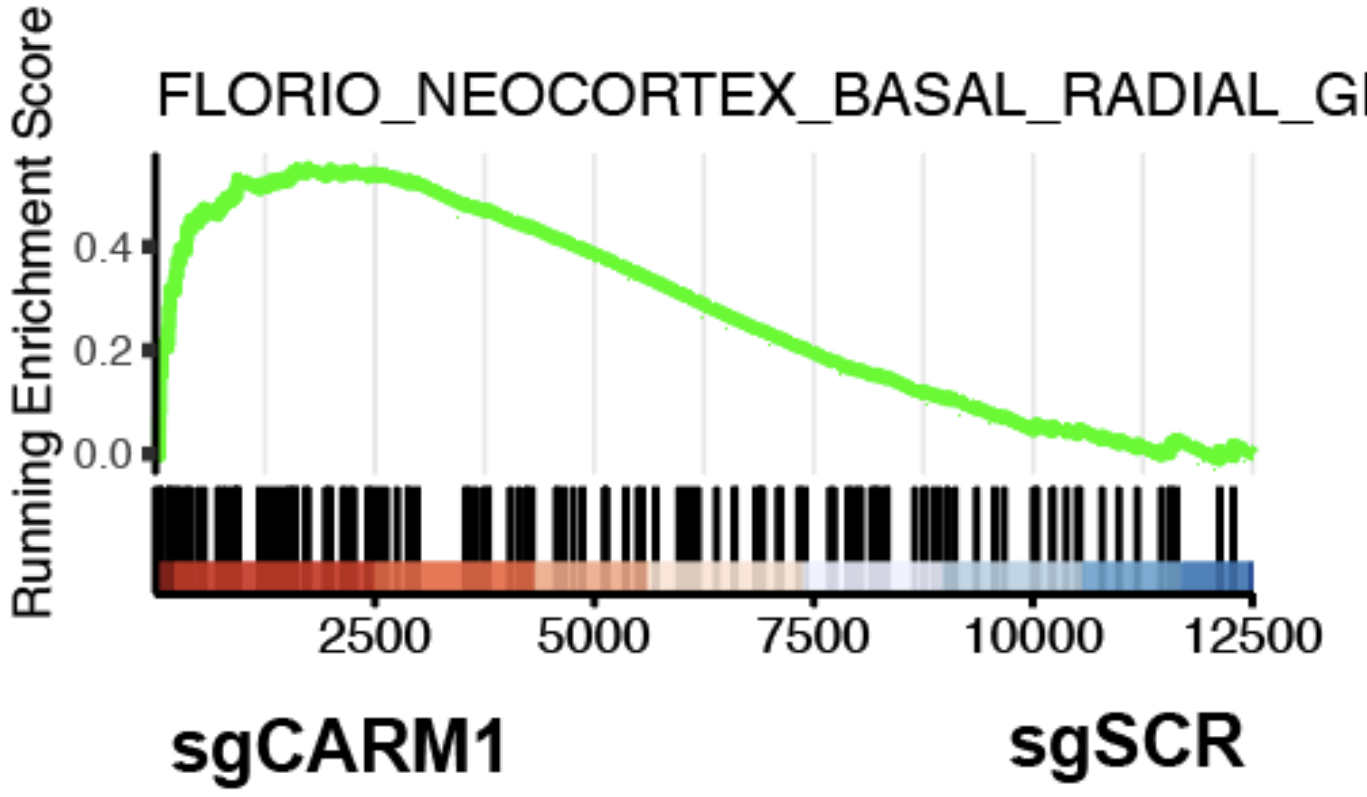

NES = 1.63
p-value = 0.002
FDR q = 0.06

B.

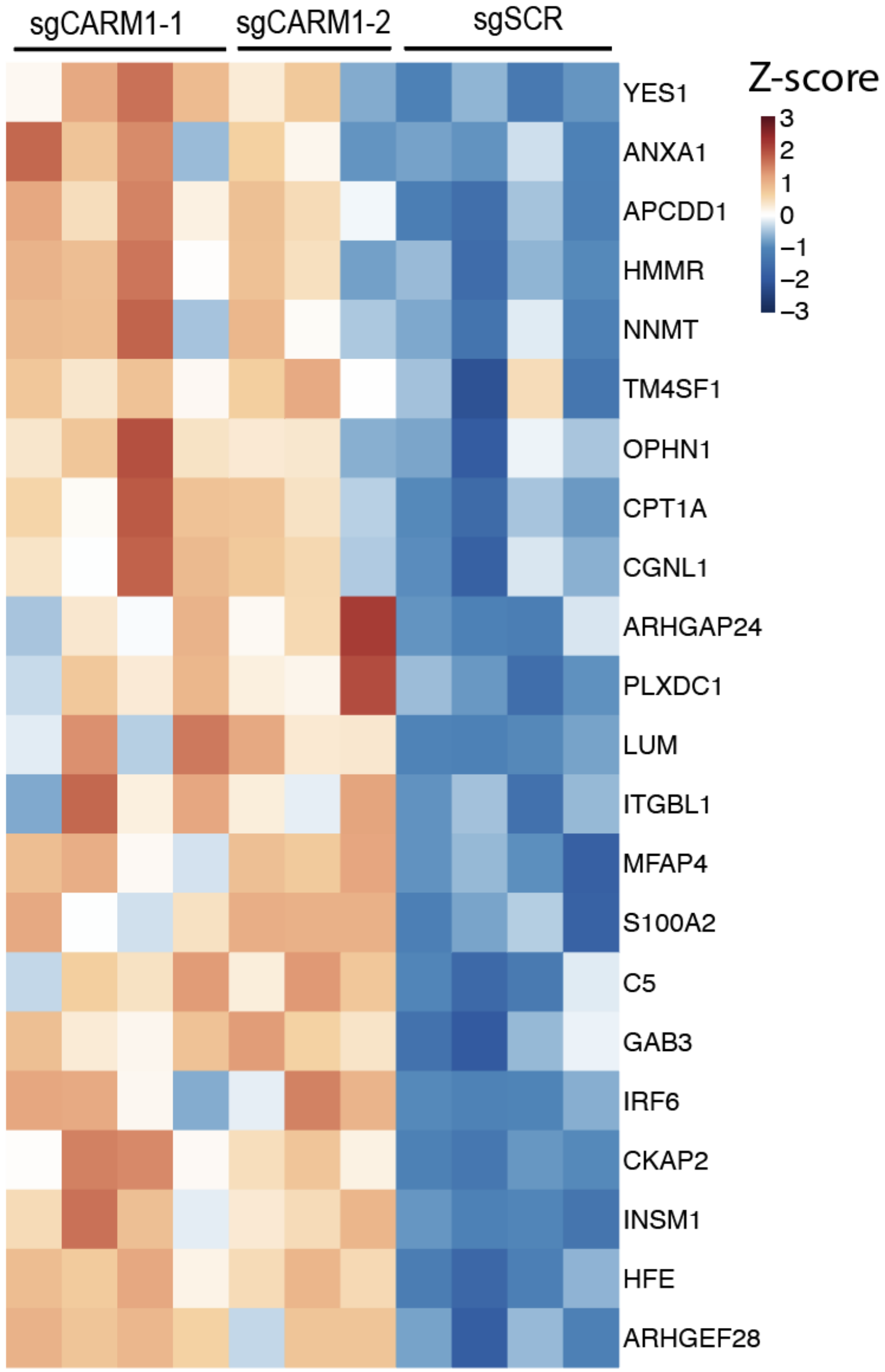

Loss of CARM1 results in a more radial-glial like transcription signature in GSCs. A) GSEA of RNA-seq and B) heatmap of differentially expressed genes found enriched in radial glial cells from panel A.

Supplementary Fig. 4

A.

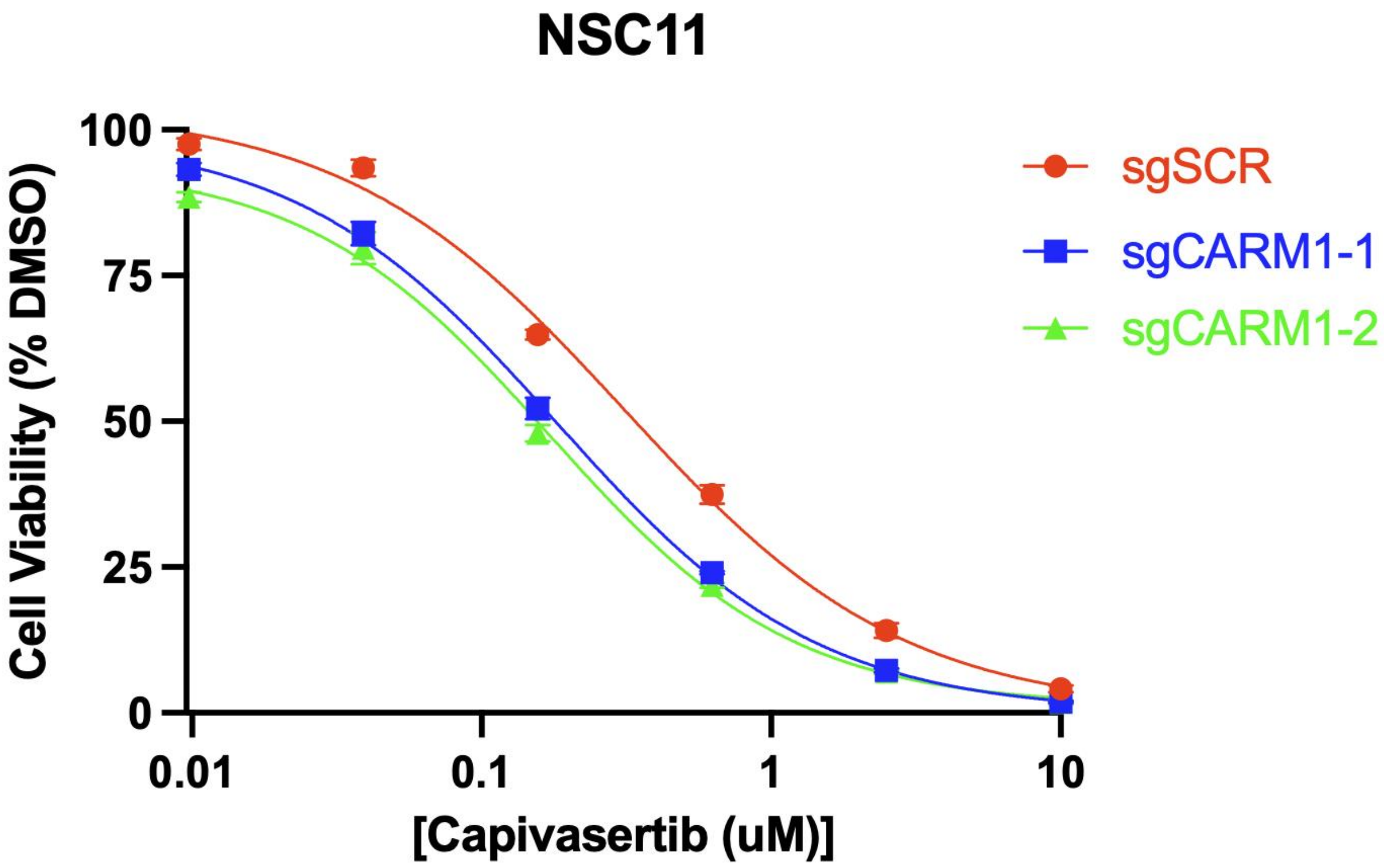

CARM1 depleted GSCs are more sensitive to AKT inhibition. A) Dose response curve of NSC11 sgSCR or sgCARM1 cells treated with the pan-AKT inhibitor, Capivasertib, n = 3. Data was calculated using Prism’s non-linear regression curve fit and data shows mean +/- SEM.

sgSCR ic50 = 312nM
sgCARM1-1 ic50 = 182nM
sgCARM1-2 ic50 = 171nM
